# Chemical Characterisation, Antidiabetic, Antibacterial, and In Silico Studies for Different Extracts of *Haloxylon stocksii* (Boiss.) Benth: A Promising Halophyte

**DOI:** 10.3390/molecules28093847

**Published:** 2023-05-01

**Authors:** Syed Nabil Raza Rizvi, Samina Afzal, Kashif-ur-Rehman Khan, Hanan Y. Aati, Huma Rao, Bilal Ahmad Ghalloo, Muhammad Nadeem Shahzad, Duraiz Ahmed Khan, Tuba Esatbeyoglu, Sameh A. Korma

**Affiliations:** 1 Department of Pharmaceutical Chemistry, Faculty of Pharmacy, Bahauddin Zakariya University, Multan 60000, Pakistan; rizvirizvi@hotmail.com; 2 Department of Pharmaceutical Chemistry, Faculty of Pharmacy, The Islamia University of Bahawalpur, Bahawalpur 63100, Pakistan; kashifur.rahman@iub.edu.pk (K.-u.-R.K.); humarao80@gmail.com (H.R.); ghall003@umn.edu (B.A.G.); shazad_sca@yahoo.com (M.N.S.); duraizahmed9@gmail.com (D.A.K.); 3 Department of Pharmacognosy, College of Pharmacy, King Saud University, Riyadh 11495, Saudi Arabia; hati@ksu.edu.sa; 4 Department of Medicinal Chemistry, College of Pharmacy, University of Minnesota, Minneapolis, MN 55454, USA; 5 Department of Food Development and Food Quality, Institute of Food Science and Human Nutrition, Gottfried Wilhelm Leibniz University Hannover, Am Kleinen Felde 30, 30167 Hannover, Germany; 6 Department of Food Science, Faculty of Agriculture, Zagazig University, Zagazig 44519, Egypt; sameh.hosny@zu.edu.eg; 7 School of Food Science and Engineering, South China University of Technology, Guangzhou 510641, China

**Keywords:** *Haloxylon stocksii*, chemical composition, enzyme inhibition, in silico study, molecular docking, amylase, glucosidase, toxicity

## Abstract

The objective of the study is to evaluate the chemical characterisation, and biological and in silico potential of *Haloxylon stocksii* (Boiss.) Benth, an important halophyte commonly used in traditional medicine. The research focuses on the roots and aerial parts of the plant and extracts them using two solvents: methanol and dichloromethane. Chemical characterisation of the extracts was carried out using total phenolic contents quantification, GC-MS analysis, and LC-MS screening. The results exhibited that the aerial parts of the plant have significantly higher total phenolic content than the roots. The GC-MS and LC-MS analysis of the plant extracts revealed the identification of 18 bioactive compounds in each. The biological evaluation was performed using antioxidant, antibacterial, and in vitro antidiabetic assays. The results exhibited that the aerial parts of the plant have higher antioxidant and in vitro antidiabetic activity than the roots. Additionally, the aerial parts of the plant were most effective against Gram-positive bacteria. Molecular docking was done to evaluate the binding affinity (BA) of the bioactive compounds characterised by GC-MS with diabetic enzymes used in the in vitro assay. The results showed that the BA of γ-sitosterol was better than that of acarbose, which is used as a standard in the in vitro assay. Overall, this study suggests that the extract from aerial parts of *H. stocksii* using methanol as a solvent have better potential as a new medicinal plant and can provide a new aspect to develop more potent medications. The research findings contribute to the scientific data of the medicinal properties of *Haloxylon stocksii* and provide a basis for further evaluation of its potential as a natural remedy.

## 1. Introduction

Herbs have been the foundation of nearly all medicinal therapy since the prehistoric era until the era of synthetic drugs in the 19th century [1]. Traditional medicines are used as an alternative therapy to treat human and animal diseases in developing countries. According to WHO, 80% of the population in emerging countries relies on herbs for disease cure [2]. Furthermore, 25% of pharmacopoeial drugs are plant derivatives or semisynthetic compounds [3]. Numerous phytochemicals have been recognised from medicinal plants and scientifically validated for their therapeutic actions [4]. Recently, there has been a significant increase in attention to discovering natural antioxidants as an alternative to synthetic antioxidants for use in medicinal materials or foods that are being restricted due to their carcinogenicity [5]. Thus, screening of new plants has remained an axis of research to find the most potent and effective drug molecule against various diseases. Medicinal plants are widely accepted in various cultures and are known to provide therapeutic benefits with minimal risks of adverse effects as compared to some synthetic products [6]. Plants comprise a diverse number of free radical scavenging moieties, such as flavonoids, anthocyanins, carotenoids, dietary glutathione, vitamins, and endogenous metabolites, and these natural moieties are rich in antioxidant potential [7,8]. Halophytes have the potential as antidiabetic agents and they have been found to possess compounds that can improve insulin sensitivity and regulate blood glucose levels [9].

Many plant species and herbs have a preservative effect, revealing the presence of antioxidant and antimicrobial components in their tissues [10]. The human body generates free radicals as part of natural physiological processes, and they can also be produced by external factors such as smoking and prolonged sun exposure. These free radicals have been associated with various metabolic and degenerative disorders, including diabetes [11]. Plants with good antioxidant potential possess antidiabetic characteristics [12]; therefore, they are becoming a point of interest for researchers as the prevalence of diabetes is rising. These plants might result in a triumph in the fight against this disorder. Antidiabetic medications can manage blood sugar levels through a variety of processes. The hepatic biosynthesis of glucose is reduced, insulin receptor sensitivity is increased, insulin secretion is stimulated, and peripheral glucose absorption is raised. Antidiabetic medications can prolong digestion and intestinal carbohydrate absorption, which aids in regulating blood sugar levels after meals [13]. Recent studies suggest that by delaying digestion, α-amylase and α-glucosidase inhibitors help to regulate blood sugar levels after meals [14]. Acarbose and miglitol, two inhibitors of α-amylase and α-glucosidase, prevented the absorption of carbohydrates from the gut [15]. The importance of in silico studies in natural product chemistry lies in their ability to save time and resources in the drug discovery process, as well as to identify new potential leads for drug development. These studies can also aid in the rational design of new analogues of natural products, to improve their bioactivity, and reduce toxicity [16].

*Haloxylon stocksii* (Boiss.) Benth belongs to the Chenopodiaceae family and is a type of halophyte plant [17]. *H. stocksii* seeds show promise as a valuable crop for arid, saline lands due to their potential as a source of edible oil and medicinal properties [18]. Its habitat is in semiarid areas and is widespread in South Asia and the Middle East. It is frequently subjected to salinity, erratic drought, and high temperatures [19]. It is a paniculately branched perennial shrub that is used as animal forage [17]. *Haloxylon stocksii* has a range of medicinal uses, including treating burns, cuts, internal ulcers, insect stings, and urinary system problems, particularly kidney and bladder stones. Additionally, it is used to alleviate symptoms associated with arthritis, joint disorders, and paralysis of the limbs [20]. Methanolic extracts of *H. stocksii* possesses anti-inflammatory, analgesic, and antiulcer properties [21]. Several extracts have been found to have hypoglycaemic, hypolipidemic, and antimicrobial properties [22,23,24]. The study of the antioxidant, antibacterial and anti-inflammation potential of this plant can reveal meaningful information regarding its use for human and medicinal purposes; however, intensive screening including antidiabetic and antioxidant potential is inevitable before its medicinal use.

To the best of the authors’ knowledge, there is no comparative study available for phytochemical characterisation, antibacterial, enzymatic inhibition, and molecular docking studies on aerial parts and root extracts of *Haloxylon stocksii*. This study has reported the phytochemical composition, antidiabetic potential, antibacterial, and molecular docking studies for both aerial parts and root extracts of *H. stocksii*. The study will provide meaningful insights into the screening of the *Haloxylon stocksii* plant as a new medicinal plant.

## 2. Results

### 2.1. Phytochemical Composition

The phytochemical characterisation of different solvent extracts of *H. stocksii* was determined by their total phenolic contents (TPC), total flavonoid contents (TFC), gas chromatography-mass spectrometry (GC-MS) analysis, and liquid chromatography-electron spray ionization mass spectrometry (LC-ESI-MS^2^) screening.

#### 2.1.1. Total Phenolic Contents (TPC)

The TPC was detected at the maximum level in the aerial methanolic extract and least in the roots dichloromethane extract (Table 1).

#### 2.1.2. Total Flavonoid Contents (TFC)

The TFC was determined in the methanolic and dichloromethane extracts of both parts, i.e., in aerial and root parts (Table 1). The TFC was significantly different in all the sample extracts. The highest TFC was exhibited by methanolic aerial parts (99.19 ± 1.14 mg of quercetin equivalent per gram of extract, mg QE/g) and lowest in the roots dichloromethane extract (54.65 ± 0.65 QE/g of extract).

#### 2.1.3. GC-MS Analysis

Due to higher phenolic contents and biological activities, methanolic extracts of aerial parts and roots were subjected to GC-MS for screening of secondary metabolites. This chemical characterisation resulted in the identification of 18 bioactive phytochemicals (Table 2). Some of these bioactive compounds are furfural; 4H-pyran-4-one, 2,3-dihydro-3,5-dihydroxy-6-methyl-; 2-furancarboxaldehyde; 5-(hydroxymethyl)-; 2-furancarboxaldehyde; 5-(hydroxymethyl)-; 1,6-anhydro-β-ᴅ-glucopyranose; phthalic acid; bis(7-methyloctyl) ester; di-n-octyl phthalate; phenol; 2,2′-methylenebis[6-(1, 1-dimethylethyl)-4-(1-methylpropyl); and γ-sitosterol. These compounds belong to the different classes, such as furans, aldehyde, pyrones, anhydrohexose, fatty acid ester.

#### 2.1.4. Phytochemical Screening of *Haloxylon stocksii* by LC-ESI-MS^2^

The methanolic extract of aerial parts was subjected to LC-ESI-MS^2^ in positive mode (Table 3, and Figures S1–S23 in the Supplementary Materials) due to its maximum total phenolic and flavonoid contents compared to other extracts. In this screening, 23 compounds were detected. Among these compounds, 18 bioactive secondary metabolites were identified with the Discoverer 3.3, and 5 were not identified with any compound library. The available toxicity profile of identified compounds is discussed in Table S1.

### 2.2. Biological Activities of Aerial and Roots Extract of H. stocksii

#### 2.2.1. Antioxidant Activities

The antioxidant activity of different sample extracts of *H. stocksii* was evaluated by DPPH, ABTS, CUPRAC and FRAP assays (Table 4). The antioxidant results of the extracts showed that aerial parts methanolic extract have maximum antioxidant activity as compared to other extracts (aerial part dichloromethane, roots methanolic, and roots dichloromethane extracts). This should be due to the highest TPC and TFC in the aerial parts methanolic extract as compared to its counterpart extract.

#### 2.2.2. Antibacterial Activity

In the current study, the antibacterial activity of the *Haloxylon stocksii* plant was determined by measuring its antibacterial activity against pathogenic bacteria. These bacteria include *Bacillus subtilis*, *Bacillus pumilus*, *Pseudomonas aeruginosa*, *Staphylococcus epidermidis*, *Escherichia coli*, *Bordetella bronchispetica*, and *Micrococcus luteus*, which are the main pathogenic and spoilage organisms. The results of the antibacterial activity of various extracts of *Haloxylon stocksii* are shown in Table 5.

#### 2.2.3. Antidiabetic Activities

**Figure 1:**
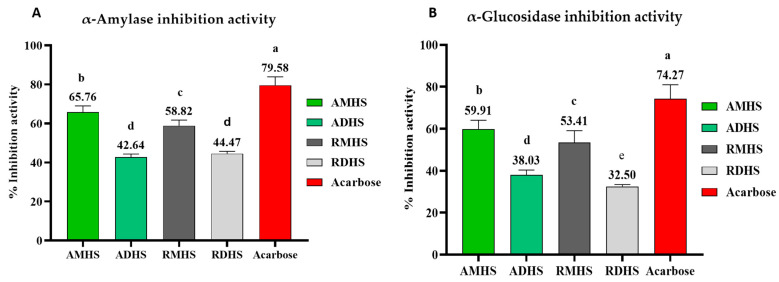
(**A**) α-amylase inhibition activity of extracts and acarbose (standard) and (**B**) α-glucosidase inhibition activity of extracts and acarbose (Standard). The superscripts a, b, c, d, and e represent significant difference (*p* < 0.05).

The potential of *H. stocksii* to alleviate diabetes was investigated in vitro by analysing its ability to inhibit two key enzymes involved in the disease, namely α-amylase and α-glucosidase. Both parts (aerial and roots) of the plant were found to exhibit significant inhibitory activity against these enzymes, indicating their potential as antidiabetic agents. Figure 1 and Table S2 shows the antidiabetic potential of this plant.

### 2.3. In silico Evaluation

In silico evaluation was done by performing molecular docking, ADME (Absorption, Distribution, Metabolism and Excretion), and toxicological studies.

#### 2.3.1. Molecular Docking (MD) for α-Amylase

**Figure 2:**
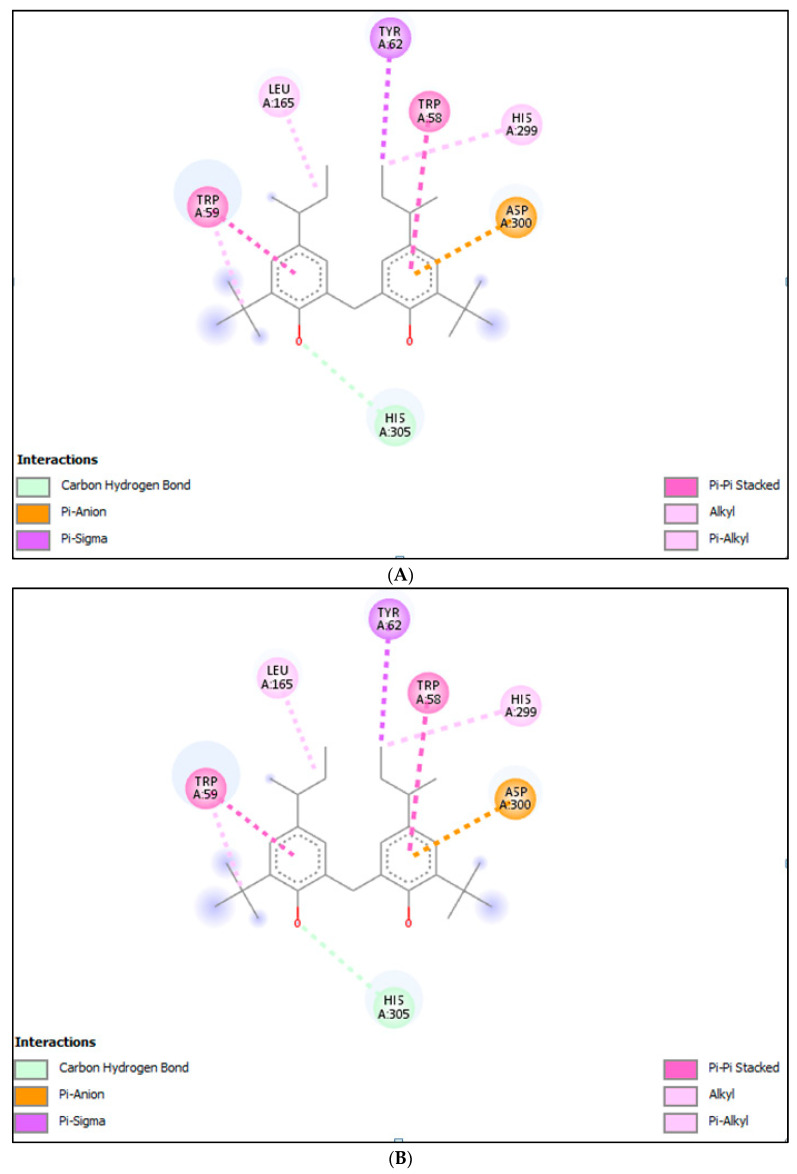

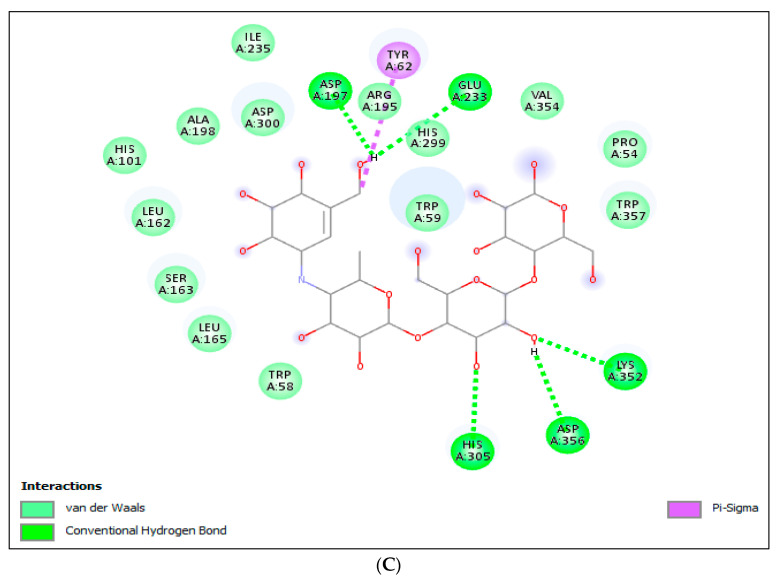
Interaction of (**A**) γ-Sitosterol, (**B**) phenol, 2,2′-methylenebis[6-(1, 1-dimethylethyl)-4-(1-methylpropyl), and (**C**) acarbose with α-amylase.

MD of GC-MS identified molecules was carried out for α-amylase receptors. In order to find out docking score, binding affinity, and interaction of different compounds at the active sites of α-amylase receptors. Amongst those, the interaction with best docked molecules in terms of binding affinity are represented in Table 4. The docking score for phenol, 2,2′-methylenebis[6-(1, 1-dimethylethyl)-4-(1-methylpropyl) and γ-sitosterol with α-Amylase receptors was (−8.5) and (−8.7), respectively. These findings reveal the potent role of *Haloxylon stocksii* in α-amylase inhibition. The details of binding affinities and interactive forces of ligands with receptors are given in Table 6 and Figure 2.

#### 2.3.2. Molecular Docking (MD) for α-Glucosidase

**Figure 3:**
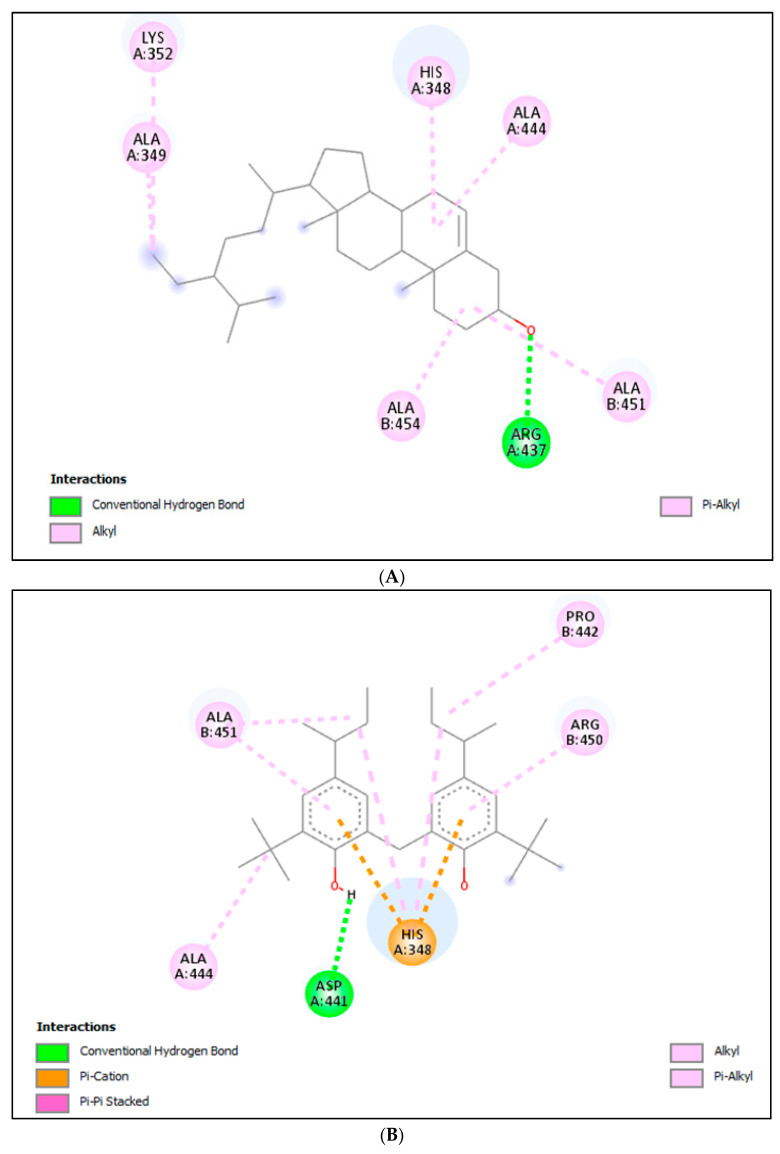

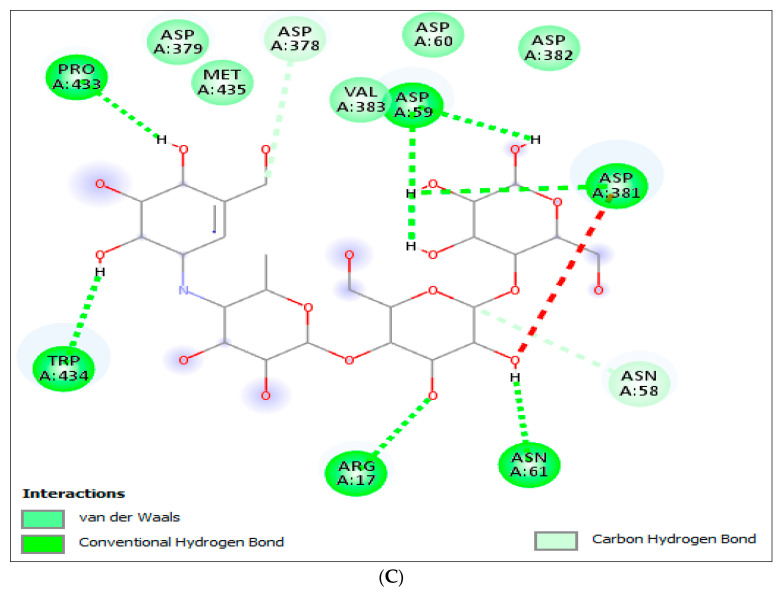
Interaction of (**A**) γ-Sitosterol, (**B**) phenol, 2,2′-methylenebis[6-(1, 1-dimethylethyl)-4-(1-methylpropyl), and (**C**) acarbose with α-glucosidase.

MD was done with α-glucosidase receptors to find out the binding score and binding interactions at active sites. For MD, molecules tentatively identified by GC-MS were selected. The binding scores for phenol, 2,2′-methylenebis[6-(1, 1-dimethylethyl)-4-(1-methylpropyl), and γ-sitosterol were −8.1 and −8.9, respectively. This reveals the key role of *Haloxylon stocksii* in α-glucosidase inhibition. The details of binding interactions, binding forces, and docking scores are given in Table 7 and Figure 3.

#### 2.3.3. ADMET Study

The SwissADME online tool was utilised to investigate the best-docked compounds in addition to the standard drug acarbose. This tool offers valuable insights into the pharmacokinetics, drug-likeness, and physicochemical properties of the compounds (Table 8). The compounds octadecanoic acid methyl ester; 4H-pyran-4-one; 2,3- dihydro-3,5-dihydroxy-6- methyl-; ethyl oleate; 1,6-anhydro-β-ᴅ-glucopyranose; and di-n-octyl phthalate have high gastrointestinal absorption. Eicosanoic acid methyl ester; phthalic acid; bis(7-methyloctyl) ester; phenol; 2,2′-methylenebis[6-(1,1-dimethylethyl)-4-(1- methylpropyl); γ-sitosterol; and acarbose have low gastrointestinal absorption. All the compounds that cannot cross blood–brain barrier have the advantage of lacking CNS adverse effects. 1,6-Anhydro-β-ᴅ-glucopyranose and the standard drug acarbose increase serum concentrations by inhibiting Pgp inhibitors. Phthalic acid, bis(7-methyloctyl) ester, and di-n-octyl phthalate can interact with the metabolism of drugs which involve cytochrome P450 3A4.

#### 2.3.4. Lipinski Rule of Five

Lipinski’s rule of five is a rule that evaluates the chemical compounds having pharmacological or biological properties that may act as orally active drugs in human beings. The definition of the Lipinski rule is that the compounds should follow the following criteria with only exception allowed of the following criterion:

1. The hydrogen bond donors should not be more than 5.
2. The hydrogen bond acceptors should not be more than 10.
3. The molecular mass should not be more than 500 daltons.
4. Lipophilicity should not exceed the value of 5.

**Figure 4:**
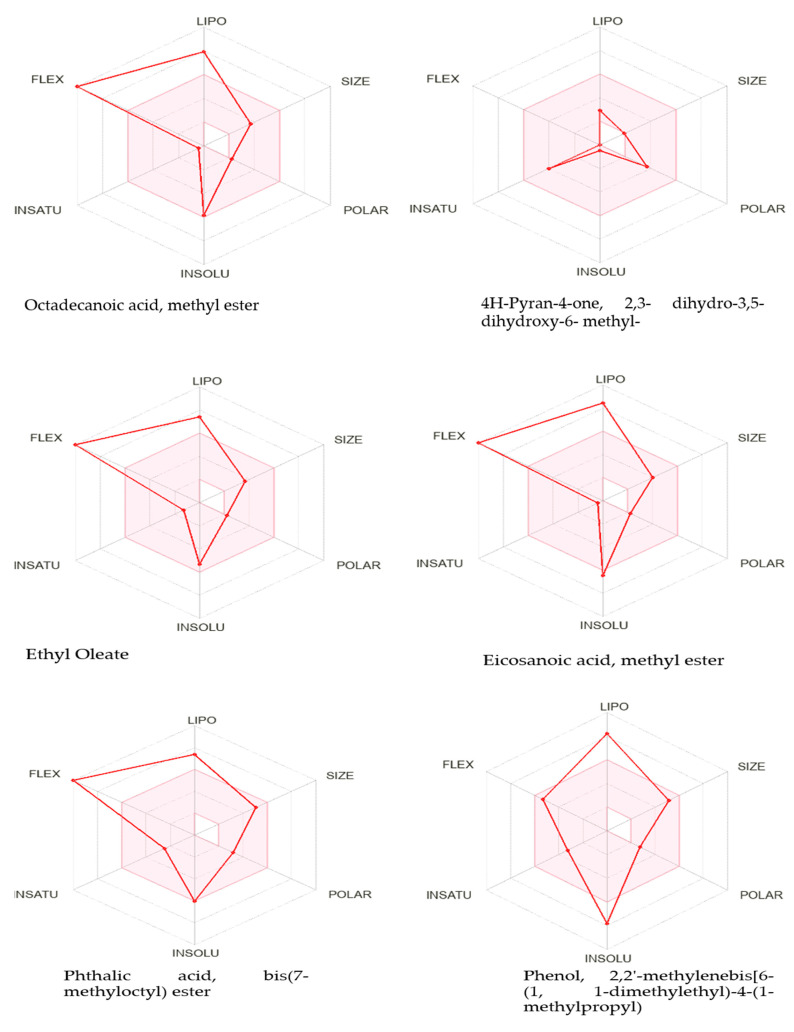

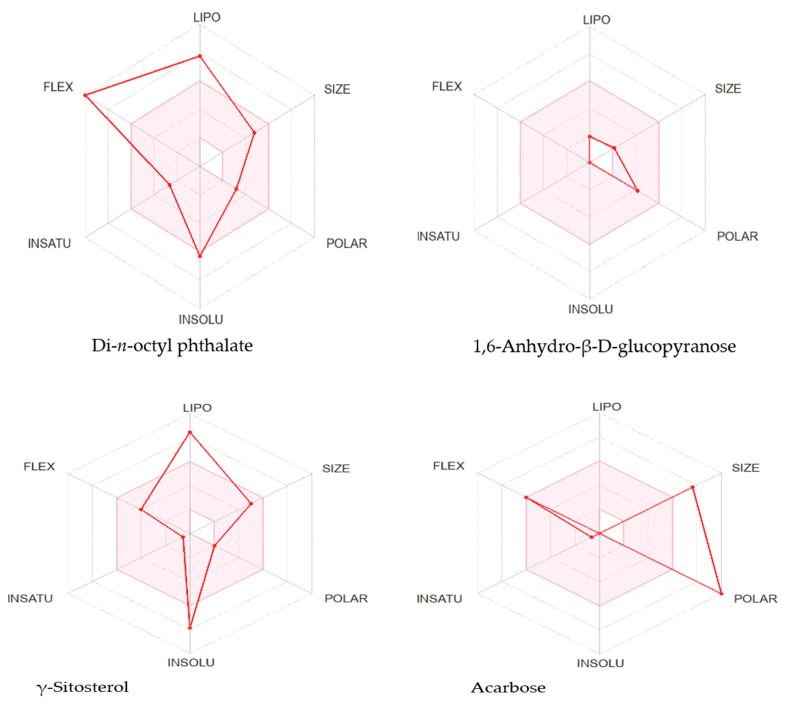
The bioavailability radar of in silico studied molecules. The pink area indicates oral bioavailability considering polarity, lipophilicity and size of the compounds.

In silico study of best-docked compounds disclosed that all the compounds have <5 hydrogen bond donors and <10 hydrogen bond acceptors. The molecular mass of all chemical compounds evaluated is less than 500 daltons. The lipophilicity value of all the observed compounds follow the rule except eicosanoic acid, methyl ester and phthalic acid, bis(7-methyloctyl) ester (Table 9 and Figure 4). So, with these observations, all the compounds can be used as oral drugs.

#### 2.3.5. Toxicity Study

During the in silico study, the toxicity profile of chemical compounds was evaluated by determining LD_50_, toxicity class, hepatotoxicity, carcinogenicity, immunotoxicity, mutagenicity, and cytotoxicity (Table 10). The toxicity findings were that all the compounds have ≥ 4 toxicity levels. This toxicity level indicates that all the compounds are slightly toxic or practically non-toxic. All the compounds except standard acarbose do not cause hepatotoxicity. Three compounds, namely phthalic acid, bis(7-methyloctyl) ester, and di-n-octyl phthalate, exhibited properties of carcinogenicity. γ-Sitosterol and acarbose displayed immunotoxicity. Only 4H-pyran-4-one, 2,3-dihydro-3,5-dihydroxy-6-methyl- exhibited mutagenicity and no chemical compound showed cytotoxicity.

## 3. Discussion

Screening of plants used for medicinal purposes has remained one of the most valuable approaches towards the development and provision of new drug candidates. The findings disclosed that there was significant difference (*p* < 0.05) among the extraction methods as well as the different plant parts, i.e., in aerial parts and roots. Briefly, it was found that AMHS samples had significantly (*p* < 0.05) high TPC and TFC compared to RMHS, which indicates that the methanol extract of aerial parts has higher TPC and TFC compared to that of the roots. Our results regarding higher TPC and TFC in aerial parts are in line with the literature that also revealed that phenolic compounds were significantly higher in aerial parts as compared to roots [45]. The difference in TPC between the roots and aerial parts could be due the presence of carotenoids in the aerial parts that are missing in the roots. Further, the effectiveness of different extraction methods was evaluated. It was observed that TPC and TFC were higher in the methanol extraction method compared to the dichloromethane extraction method. In addition, the higher TPC and TFC levels in the aerial parts indicate the presence of polyphenolic secondary metabolites, which may be a result of the environmental stress factors that the aerial parts face. This could be the reason that aerial parts face more stress against the environmental factors, which produce more antioxidant compounds as compared to roots, agreeing well with previous studies [46,47]. Peerzada, Khan et al. determined the TPC and TFC (262.6 ± 7.49 mg GAE/g and 79.86 ± 6.02 mg QE/g, respectively) in methanolic extract of the whole plant [48]. These high values may be due to difference in habitat of plant or due to synergistic effect of both aerial parts and roots.

GC-MS analysis is a prevailing analytical technique used in the identification of the components present in plant extracts [49]. This technique is particularly useful in the field of natural product chemistry, where plant extracts are commonly used as a source of bioactive secondary metabolites [50]. One of the main advantages of GC-MS analysis of plant extracts is its high sensitivity and specificity. The technique can detect and identify components present in trace amounts, allowing for the detection of compounds that may have important biological activities even at low concentrations [51]. Additionally, mass spectrometry is used for the characterisation of the molecular weight and structure of the identified components, which is critical for understanding their biological activities and potential therapeutic applications.

Liquid chromatography-mass spectrometry (LC-MS) profiling of plant extracts is an effective analytical approach for identifying the numerous chemical components found in plant extracts. It gives a thorough breakdown of the extract’s phytochemical makeup, including flavonoids, alkaloids, terpenoids, and other bioactive substances. The identification of unidentified chemicals in the extract via LC-MS analysis can also aid in the discovery of novel natural products with potential medicinal uses. In general, LC-MS profiling of plant extract is a useful tool for characterising chemicals obtained from plants, and it can help create new medications and functional food ingredients. The LC-MS screening resulted in the identification of amines, aromatic ketones, phenyl amines, flavonoids, alkaloids, organophosphate esters, fatty acids, and glycerolipids.

Antioxidant properties are of great importance as they determine the suitability and applicability of these plants for medicinal use. Researchers are seeking new plants to assess their effectiveness, as they believe that these are the best alternatives, with the fewest side effects, to medicines. Plant extracts have been recognized as a rich source of antioxidants, which have played a critical role in the prevention of various illnesses, including cancer, cardiovascular diseases, diabetes, and neurodegenerative disorders [52]. The antioxidant property of plant extracts is primarily due to their content of polyphenolic compounds, likely flavonoids, phenolics, and tannins [53]. Numerous studies have reported the antioxidant properties of plant extracts from various sources, including roots, vegetables, and aerial parts [54,55]. Overall, plant extracts are a promising source of antioxidants that could have potential health benefits, although further studies are needed to better understand their bioavailability and potential therapeutic applications. A study conducted by Yaseen et al. (2020) investigated the total antioxidant activity of the acetone, chloroform, acetic acid and propranolol extracts of *Haloxylon stocksii* [56]. The literature review of some bioactive phytoconstituents, identified with GC-MS and LC-MS, possess antioxidant activities. These bioactive secondary metabolites include triacontanoic acid [57], octadecanoic acid [44], γ-sitosterol [58], and fraxetin [59]. There is no antioxidant comparative study of the aerial and roots part of *H. stocksii* available in literature by utilising methanol and dichloromethane as extraction solvents. This comparative study is the first one that reports the antioxidant potential of *H. stocksii* for different parts and with different extraction methods.

The antibacterial potential of plants has great importance regarding their use in medicine as this property mainly involves the reduction of pathogenic microbes [60]. The results of the current study exhibited that aerial parts methanolic extract (AMHS) is more active to Gram-positive bacteria, and roots methanolic extract (RMHS) is more active against Gram-negative bacterial strains. These antibacterial activities may be due to the presence of several compounds which have antimicrobial activity. These compounds include 2-furancarboxaldehyde; 5-(hydroxymethyl)- [28]; 1,6-anhydro-β-ᴅ-glucopyranose [29]; hexadecanoic acid; methyl ester; 9-octadecenoic acid; methyl ester [52]; heptadecanoic acid; 16-methyl ester [35]; phthalic acid; bis(7-methyloctyl) ester [41]; hordenine [61]; fraxetin [62]; piperine [63]; stearamide [64]; and erucamide [65]. Wahab et al. reported the antibacterial activity against some Gram-positive and Gram-negative bacterial strains. Their results revealed that the methanolic extract of the whole plant showed good antibacterial activity against tested strains and was maximum against *S. typhi* [66]. *Anabasis aretioides,* a member of Chenopodiaceae, and showed the best activity against *Staphylococcus aureus* [67].

Type 2 diabetes (T2D) is a prevalent health condition worldwide that results in high blood sugar levels and abnormal carbohydrate metabolism. This disease is a significant contributor to illness and death, and it also places a substantial economic burden on societies [68]. The *H. stocksii* extracts were evaluated in vitro for antidiabetic activity (α-amylase and α-glucosidase inhibition). The aerial parts extracts were more effective compared to that of the roots. When we compare the two solvents, the methanol extract was more effective as compared to the dichloromethane extract. The higher antidiabetic potential of the methanolic extraction method could be due to the higher TPC of this method. Truong et al. demonstrated that the methanolic extraction method gives maximum extraction yield, highest phenolics and flavonoid contents, and antioxidant activity as compared with ethanol, chloroform, acetone, and dichloromethane extraction methods [69]. In addition, higher polyphenols are found to be effective in diabetes management. The antidiabetic potential of this plant is attributed due to the presence of some bioactive phytocompounds which were identified with GC-MS and LC-MS, such as β-sitosterol [51]; phenol; 2,2′-methylene bis[6-(1,1-dimethylethyl)-4-(1-methylpropyl) [55]; hordenine [70]; and piperine [63]. The compounds having good antioxidant activities are also responsible for antidiabetic activity [56]. The aqueous extract of *Chenopodium botrys* exhibited α-amylase and α-glucosidase inhibition activity [71]. *Atriplex halimus* L. significantly increases body weight and decreases blood glucose and hepatic levels in the rats [72]. The results of in vitro antidiabetic activities of plants endorse that oral administration of these extracts, especially aerial parts extracts in non-insulin-dependent diabetes, can reasonably reduce postprandial blood glucose levels; however, future studies, especially in vivo studies, are necessary to confirm their effectiveness.

In silico studies of *Haloxylon stocksii* have been gaining attention due to their cost-effectiveness and ability to provide insights into the potential biological activities of the plant’s bioactive compounds. Several studies have used computational approaches such as molecular docking, ADME, and toxicological studies to predict the binding affinity and activity of *Haloxylon stocksii*’s bioactive compounds with various therapeutic targets [55]. In silico studies have identified several compounds with potential antidiabetic, anti-inflammatory, and anticancer activities, such as stigmasterol and β-sitosterol. These studies have also helped to elucidate the mechanism of action of the plant’s bioactive compounds and have guided further experimental studies [73]. Therefore, future studies should aim to combine in silico and in vitro/in vivo experiments to further validate the potential therapeutic activities of *Haloxylon stocksii*’s bioactive compounds.

Overall, these studies suggest that *Haloxylon stocksii* could be a potential source of bioactive compounds, natural antioxidants, antibacterial, and antidiabetic and that further research is needed to better understand the bioactive compounds present in the plant extract and their potential therapeutic applications.

## 4. Materials and Methods

### 4.1. Plant Identification and Sample Collection

The roots and aerial parts of the plant were collected from sandy cliffs of Tehsil Choti Zarieen District Dera Ghazi Khan in August 2020 Punjab, Pakistan. The plant was identified as *Haloxylon stocksii* by Zafar Ullah Zafar, Institute of Pure and Applied Biology, Bahauddin Zakariya University Multan, Pakistan (BZU). Voucher no. http://www.theplantlist.org/tpl1.1/record/tro-50307719 (accessed on the 20 May 2020) was given to that plant.

### 4.2. Extract Preparation of the Plant Material

Firstly, the plant was washed, and shade-dried in two separate portions (i.e., aerial parts and roots) for 6 weeks at 25 °C. Both the plant parts were pulverized roughly to a coarse powder and the extraction of both powdered materials was done by simple maceration procedure separately in dichloromethane (DCM) and methanol (the ratio between powder and solvent was 1:3). Then, filtration was done after 24 h. This procedure was repeated four times with both these solvents. DCM and methanol extracts were dried under reduced pressure in a rotary evaporator (Buchi, Flawil, Switzerland). The DCM extract of roots (50.0 g) and aerial parts (30.8 g) were collected in sample bottles and assigned codes RDHS and ADHS, respectively. Methanol resulted in the yield of roots extract (70.4 g) and aerial parts extract (52.0 g). These dried extracts were collected in the sample bottles and assigned codes RMHS and AMHS, respectively.

### 4.3. Phytochemical Composition

#### 4.3.1. Estimation of Total Phenolic Content

The determination of total phenolic contents (TPC) and total flavonoid contents (TFC) were done using the Folin–Ciocalteu (FC) and aluminium trichloride colorimetric methods, respectively [44].

#### 4.3.2. GC-MS Analysis

GC-MS screening was done with an Agilent 6890 series and Hewlett Packard 5973 ground sensor. The HP-5MS column (Santa Clara, CA, USA) with 30 m length × 250 µm diameter × 0.25 µm film thickness was used. The temperature of the injection was set at 220 °C up to 240 °C, while the temperature of the oven was programmed from 60 °C to 246 °C at a rate of 3 °C/min. Pure He (helium) gas was used as a carrier. A volume of 1.0 μL of the reconstituted sample extract was injected. The temperature was set between 50 °C and 150 °C at a rate of 3 °C/min, and then raised to 300 °C at a rate of 10 °C/min. The identification of bioactive compounds was performed by tentatively identifying peaks using the NIST 2011 library scanning ranging from 35 to 600 *m/z* [74].

#### 4.3.3. Phytochemical Screening of *Haloxylon stocksii* by LC-ESI-MS2

The aerial parts methanolic extract of *H. stocksii* was subjected to LC-ESI-MS2 analysis for qualitative determination of the secondary metabolites. The sample extract was dissolved in methanol of LC-MS grade. This solution was filtered and then injected by direct syringe method at the flow rate of 5 µL/min. The system consists of a column with dimensions 250 × 2.0 mm ODS-VP C18, 5 µm. The mobile phase used in liquid chromatography comprises the solvent A (0.1 % formic acid) and solvent B (methanol with 0.1 % formic acid). Gradient method of elution with 5 % B (0–5 min), 5–45 % B (90–100 min), 45–5% B (100–101 min) was then used. Afterwards, it was evaluated by Tandem Mass Spectrometry and LTQ XL linear Ion Trap Mass Spectrophotometer having an Electron spray ionization interface. The compounds were identified with Compound Discoverer 3.3. The sample was scanned at positive mode and the scan range of mass was m/z 50–2000. The voltage used was 4.8 KV, and capillary voltage used was 23 V during the analysis. The temperature of the capillary was 350 °C during the scan.

### 4.4. Biological Profiling

#### 4.4.1. Antioxidant Activities

The roots and aerial parts of the *Haloxylon stocksii* plant were evaluated for antioxidant potential through different antioxidant assays which include the determination of radical scavenging activity and reducing power. Trolox was used as standard, and results were expressed as milligrams of Trolox equivalents per gram of dry extract (mg TE/g extract). Control was prepared by the same procedure without adding plant extract.

##### Radical Scavenging Activity

Aerial parts and roots of the plant were analysed for the evaluation of scavenging potential and were evaluated using 1,1-diphenyl-2-picrylhydrazyl (DPPH) and 2,2-azinobis 3-ethylbenzothiazoline-6-sulfonic acid (ABTS) assays. The procedures for above mentioned assays were in line with the literature [75].

1. DPPH Assay

For this radical scavenging assay, the volume of 50 µL stock solutions of the extracts of both parts of the plants were mixed with 200 µL of DPPH solution (0.267 mM). After, these mixtures were incubated in a 96-well microtiter plate at 25 °C for 30 min (in darkness). The absorbance of samples was measured by a microplate reader (BioTek Synergy HT, Winooski, Vermont, USA) at wavelength 517 nm. The blank solution was also assessed according to this procedure.

2. ABTS Assay

For this reducing power assay, stock solutions of the extracts of both parts of the plants were prepared and diluted with ABTS^+^ solution until absorbance reached 0.700 ± 0.02 at 734 nm. The volume of 100 µL of extract solutions were mixed with 200 µL of ABTS^+^ solution in 96-well microplate. This mixture was incubated at 25 °C for 30 min. After this, absorbance of both solutions was measured at wavelength 734 nm. The control solution was also assessed according to this procedure.

##### Reducing Power Assays

Both parts of the *H. stocksii* plant were assessed for their reducing capacity by utilising direct reducing antioxidant Cupric Reducing Antioxidant Capacity (CUPRAC) and Ferric Reducing Antioxidant Power (FRAP). Both mentioned assays were performed according to the procedures mentioned in the literature [76].

1. CUPRAC Assay

For performing the CUPRAC assay, stock solutions of the extracts of both parts of the plant were prepared and added to reaction mixture containing neocuproine (200 µL, 7.5 mM), cupric chloride (200 µL, 10 mM), and ammonium acetate buffer (200 µL, 1 M, pH 7.0)]. After this, both solutions were incubated at 25 °C for 30 min, and absorbance was measured at 450 nm. The control solution was also assessed according to this procedure.

2. FRAP Assay

For performing the FRAP assay, stock solutions of the extracts of both parts of the plants were prepared and added to acetate buffer in 40 mM HCl and ferric chloride (20 mM) in a final concentration with the ratio of 10:1:1 (*v*/*v*/*v*). Absorbance was measured at 593 nm after the incubation for 30 min at 25 °C. Furthermore, a blank solution was also assessed according to this procedure.

#### 4.4.2. Antibacterial Activities

Antibacterial activities of the *Haloxylon stocksii* plant aerial parts and roots were assessed against seven bacterial stains. Three Gram-negative stains (*Pseudomonas aeruginosa* ATCC 9027*, Escherichia coli* ATCC 25922, and *Bordetella bronchiseptica* ATCC 7319) and four Gram-positive strains (*Bacillus subtilis* ATCC 1692*, Bacillus pumilus* ATCC 13835*, Staphylococcus epidermidis* ATCC 8724*,* and *Micrococcus luteus* ATCC 4925) of bacteria. These strains were obtained from Drug Testing Laboratory, Lahore (DTL). Co-amoxiclav was used as standard in this assay. The 24 h old cultures of all seven strains were taken, prepared inoculum by taking few colonies of each bacterium and adding them in test tubes containing broth medium. Tubes were incubated overnight, and colonies were diluted to a cell density of 10^6^ CFU/mL (colony forming unit).

##### Disc Diffusion Method

This method was used to estimate the antibacterial activity of common infection-causing bacterial strains from the aerial parts and roots of the *Haloxylon stocksii* plant. The results were measured as zones of inhibition. Solutions were prepared in 10% of DMSO. These solutions were sterilised by filtering with a sterile membrane filter. After this Petri dishes with Mueller Hinton agar were prepared and inoculated with the seven bacterial strains. Positive and negative control discs were made using 10% DMSO and co-amoxiclav (10 µg/disc), respectively. These Petri dishes were incubated at 37 °C for 24 h. Triplicate experiments were performed to minimize the error. The zone of inhibition was measured in millimetres (mm), and greater than 6 mm was considered for antibacterial activity [52].

#### 4.4.3. In vitro Antidiabetic Potential

For the estimation of the in vitro antidiabetic potential in aerial parts and root extracts of *H. stocksii*, α-amylase and α-glucosidase inhibition assays were performed.

- α-Amylase inhibitory assay:

The α-amylase inhibition assay of methanolic and DCM extracts of aerial parts and roots was performed according to the method described in the literature with minor changes [73]. The volume of 0.5 mL extracts of both parts was mixed with 0.5 mL of α-amylase solutions with 0.02 M of sodium phosphate buffer (pH 6.9) this mixture was kept for incubation at 25 °C for 10 min, and then the reaction was terminated by adding 1 mL of the colouring agent dinitrosalicylic acid. After this, these test tubes were heated in a warm bath at 100 °C for 5 min. After cooling the reaction mixture till 25 °C, it was diluted with 10 mL of deionized water, and the absorbance of blank and control samples was measured at 540 nm. Acarbose was used as a standard drug. The following equation measured the inhibition of α-amylase.

$$\%\;inhibition\;of\;\alpha -amylase=\left(Abs_{Control}-Abs_{Sample}\right)/\left(Abs_{Control}\right)\times 100$$

- α-Glucosidase inhibitory assay:

For determination of the antidiabetic potential crude methanolic and DCM extracts of both aerial and root, parts were also analysed using α-glucosidase inhibitory assay [13]. For this analysis, 1 mL of each extracted part was mixed with 100 µL of phosphate buffer of pH 5.9. Then, 50 µL of enzyme α-glucosidase was added and kept at 37 °C for 10 min for incubation. The volume of 50 µL of substrate, i.e., *p*-nitrophenyl-α-ᴅ-glyco-pyranoside (pNPG) was added to the reaction mixture and the mixture was kept for incubation for a further 30 min at 35 °C. After complete incubation, the absorbance of all samples was measured at 405 nm. The % inhibition was measured by the following formula.

$$\%\;inhabition\;of\;\alpha -glucocidase=1-\left(Abs_{Sample}-Abs_{Control}\right)\times 100$$

### 4.5. In-Silico Evaluation

In silico evaluation was performed by molecular docking, ADME, and toxicity studies.

#### 4.5.1. Molecular Docking Study

Molecular docking plays a key role in computer-aided drug designs and the development of molecular biology. For molecular docking in the current study, various software tools were employed, such as Autodock Vina, MGL Tools, PyRx, Babel and Discovery Studio. The enzymes α-amylase (PDB DOI: 10.2210/pdb1SMD/pdb, accessed on 7 August 2022) and α-glucosidase (PDB DOI: 10.2210/pdb5ZCB/pdb, accessed on 7 August 2022) were downloaded from Protein Data Bank [77]. The enzymes were prepared by Discovery Studio (Discovery Studio 2021 client). The GC-MS identified compounds along with acarbose (standard drug) were used as ligands and the SDF format of these ligands was downloaded from PubChem. Open Babel was used for the preparation of Ligand. Both prepared receptor and ligands were loaded to vina, embedded in PyRx. These were placed in the active area of the enzyme and the evaluation of outcomes was carried out by using Discovery Studio Visualizer [10].

#### 4.5.2. ADMET Analysis

The ADMET analysis of the compounds were performed with online SwissADME database (http://www.swissadme.ch/, accessed on 15 October 2022) [78].

#### 4.5.3. Toxicity Evaluation

The toxicity of in silico studied compounds were performed by the online tool PROTOX II (https://tox-new.charite.de/protox_II/, accessed on 13 October 2022) [73].

## 5. Conclusions

The exploration of new plants for drug development is a promising approach in the search for more effective therapies. In this study, *Haloxylon stocksii* was evaluated for its potential in various parameters including polyphenol contents, antioxidant, antibacterial, and antidiabetic properties. The antidiabetic potential of methanolic extracts was better than that of dichloromethane extracts. The results demonstrate that aerial parts extracts are more effective against-Gram-positive bacteria and root extracts showed more activity against Gram-negative bacteria. These findings suggest that *H. stocksii* may have significant potential for drug development, particularly in the treatment of infections and metabolic diseases; however, further investigation, including in vitro and in vivo cytotoxicity assays to evaluate the safety of the identified compounds is necessary to fully validate these findings before considering *H. stocksii* as a viable candidate for drug development. Nonetheless, these results may represent a promising milestone in the medical industry’s search for novel and effective therapies.

## Figures and Tables

**Table 1:** TPC and TFC values for different extracts (dry weight) of *H. stocksii*.

Extract	TPC (mg GAE/g Extract)	TFC (mg QE/g Extract)
AMHS	119.58 ± 2.45 ^a^	99.19 ± 1.14 ^a^
ADHS	102.65 ± 1.79 ^b^	87.54 ± 0.73 ^b^
RMHS	91.54 ± 2.65 ^c^	65.65 ± 0.65 ^c^
RDHS	77.65 ± 1.91 ^d^	54.65 ± 0.84 ^d^

Results are expressed as mean ± standard deviation. “AMHS” aerial parts methanolic *H. stocksii* extract; “ADHS” aerial parts dichloromethane *H. stocksii* extract; “RMHS” roots methanolic *H. stocksii* extract; and “RDHS” roots dichloromethane *H. stocksii* extract. “GAE/g extract” Gallic acid equivalent per gram of extract, “QE/g extract” Quercetin equivalent per gram of extract, the superscripts a, b, c, and d represent significant difference (*p* < 0.05).

**Table 2:** GC-MS analysis of aerial parts and roots of *H. stocksii*.

Sr. No.	Compound Name	Plant Part	Molecular Formula	M.W. (g/moL)	Rt (min)	Percentage Area (%)	Chemical Class	Biological Activity
1	Furfural	A, R	C_5_H_4_O_2_	96.08	2.73	2.43	Furan aldehyde	Antimicrobial andantioxidant [25]
2	2-Furancarboxaldehyde, 5-methyl-	A, R	C_6_H_6_O_2_	110.1	3.65	2.38	Furans and aldehyde	Antimicrobial and antioxidant [26]
3	4H-Pyran-4-one, 2,3-dihydro-3,5-dihydroxy-6-methyl-	A, R	C_5_H_6_O_4_	130.10	5.91	3.80	Pyrones	Antimicrobial and antioxidant [27]
4	2-Furancarboxaldehyde, 5-(hydroxymethyl)-	A, R	C_6_H_6_O_3_	126.1	6.64	24.05	Aryl-aldehude	Antioxidant and antibacterial [28]
5	1,6-Anhydro-β-ᴅ-glucopyranose	R	C_6_H_10_O_5_	162.14	9.22	5.95	Anhydrohexose	Antibacterial [29]
6	Hexadecanoic acid, methyl ester	A, R	C_17_H_34_O_2_	270.5	10.52	1.13	Fatty acid ester	Antibacterial and antioxidant [30]
7	Pentadecanoic acid, 14-methyl-, methyl ester	R	C_17_H_34_O_2_	270.5	10.53	1.70	Fatty acid methyl ester	Antioxidant and antimicrobial [31]
8	9-Octadecenoic acid, methyl ester	A, R	C_19_H_36_O_2_	296.5	11.98	1.75	Fatty acid ester	Antibacterial, antioxidant, and anti-inflammatory [32]
9	Octadecanoic acid, methyl ester	R	C_19_H_38_O_2_	298.5	12.16	0.29	Fatty acid methyl ester	Cytotoxicity, antioxidant activity, and anti-inflammatory [33,34]
10	Heptadecanoic acid, 16-methyl-methyl ester	A	C_19_H_38_O_2_	298.5	12.15	0.08	Fatty acid ester	Antibacterial and antioxidant [35]
11	Ethyl Oleate	A, R	C_20_H_38_O_2_	310.5	12.51	0.31	Fatty acid ethyl ester	Antioxidant and antimicrobial [36]
12	Eicosanoic acid, methyl ester	A, R	C_21_H_42_O_2_	326.6	13.83	0.03	Fatty acid ester	Antimicrobial and antioxidant [37]
13	1-Octadecene	R	C_18_H_36_	252.5	13.49	0.23	Octadecene	Antimicrobial [38]
14	Phenol, 2,2′-methylenebis[6-(1, 1-dimethylethyl)-4-(1-methylpropyl)	A	C_29_H_44_O_2_	424.7	14.89	0.05	Phenol	
15	Docosanoic acid, methyl ester	A	C_23_H_46_O_2_	354.6	15.87	0.04	Fatty acid methyl ester	Antioxidant [39]
16	Di-n-octyl phthalate	A, R	C_24_H_38_O_4_	390.6	16.16	0.21	Phthalate ester	Anticancer and antioxidant [40]
17	Phthalic acid, bis(7-methyloctyl) ester	A	C_26_H_42_O_4_	418.6	19.77	0.18	Phthalate ester	Antioxidant and antimicrobial [41]
18	γ-Sitosterol	A	C_29_H_50_O	414.7	21.14	0.25	Phytosterols	Analgesic, antioxidant, antidiabetic, andantibacterial [42,43,44]

A: aerial parts of *H. stocksii*. R: roots of *H. stocksii*.

**Table 3:** LC-ESI-MS^2^ profiling of methanolic extract of aerial parts.

Sr. No.	Retention Time (Minutes)	M/Z	Compound Name	Molecular Formula	Molecular Mass	Chemical Class
1	3.504	152.107	N-Methyltyramine	C_9_H_13_NO	151.099	Amines
2	3.505	121.065	Acetophenone	C_8_H_8_O	120.057	Aromatic ketones
3	4.004	166.122	Hordenine	C_10_H_15_NO	165.115	Phenyl amines
4	10.477	420.144	Methyl 2-(4-oxo-3-((4-(p-tolyl)thiazol-2-yl)methyl)-3,4-dihydrophthalazin-1-yl)acetate	C_23_H_21_N_3_O_3_ S	419.137	Acetamides
5	11.821	209.044	Fraxetin	C_10_H_8_O_5_	208.037	Flavonoids
6	15.267	223.059	6,8-Dihydroxy-7-methoxy-3-methyl-1H-isochromen-1-one	C_11_H_10_O_5_	222.053	Flavonoids
7	16.846	300.122	Unknown	C_17_H_17_NO_4_	299.115	
8	17.395	247.179	nor-3-Methylfentanyl	C_15_H_22_N_2_O	246.173	Opioids
9	19.106	314.138	Moupinamide	C_18_H_19_NO_4_	313.131	Amides
10	27.591	343.117	2-(2,6-Dimethoxyphenyl)-5,6-dimethoxy-4H-chromen-4-one	C_19_H_18_O_6_	342.109	
11	29.578	286.143	Piperine	C_17_H_19_NO_3_	285.136	Alkaloids
12	38.689	399.251	Tris(2-butoxyethyl) phosphate	C_18_H_39_O_7_P	398.243	Phosphate esters
13	43.688	403.232	Acetyl tributyl citrate	C_20_H_34_O_8_	402.225	Organophophate esters
14	45.134	352.321	Unknown	C_22_H_41_NO_2_	351.314	
15	45.755	309.242	Unknown	C_19_H_32_O_3_	308.235	
16	46.749	256.263	Hexadecanamide	C_16_H_33_NO	255.256	Fatty acid amides
17	48.076	354.337	Unknown	C_22_H_43_NO_2_	353.329	
18	48.144	331.284	1-Palmitoylglycerol	C_19_H_38_O_4_	330.277	Glycerolipid
19	49.561	191.143	(1S)-Tricyclo[7.3.1.0~2,7~]tridec-2(7)- en-13-one	C_13_H_18_O	190.136	Cyclic ketone
20	52..221	284.294	Stearamide	C_18_H_37_NO	283.287	Long-chain fatty acid
21	55.801	338.342	Erucamide	C_22_H_43_NO	337.334	Long-chain fatty acid
22	55.152	394.347	Unkown	C_28_H_43_N	393.339	
23	56.318	122.096	N,N-Dimethylaniline	C_8_H_11_N	121.089	Aromatic amines

“Unknown” Not identified with Discoverer 3.3.

**Table 4:** DPPH, ABTS, CUPRAC, and FRAP values for different extracts (dry weight) of *H. stocksii*.

Extract	DPPH (mg TE/g Extract)	ABTS (mg TE/g Extract)	FRAP (mg TE/g Extract)	CUPRAC (mg TE/g Extract)
AMHS	145.45 ± 2.94 ^a^	98.07 ± 3.47 ^a^	231.76 ± 7.69 ^a^	410.08 ± 10.51 ^a^
ADHS	121.65 ± 3.4 ^b^	66.65 ± 2.93 ^b^	192.27 ± 5.7 ^b^	367.42 ± 8.35 ^b^
RMHS	93.41 ± 1.99 ^c^	68.07 ± 1.15 ^b^	168.67 ± 3.81 ^c^	332.65 ± 7.91 ^c^
RDHS	74.98 ± 1.28 ^d^	52.98 ± 0.82 ^c^	145.64 ± 2.6 ^d^	296.12 ± 5.84 ^d^

Results are expressed as mean ± standard deviation. “DPPH” 1,1-diphenyl-2-picrylhydrazyl; “ABTS” (2,2′-azino-bis(3-ethylbenzothiazoline-6-sulfonic acid)); “FRAP” Ferric ion reducing antioxidant power; “AMHS” aerial parts methanolic *H. stocksii* extract; “ADHS” aerial parts dichloromethane *H. stocksii* extract; “RMHS” roots methanolic *H. stocksii* extract; and “RDHS” roots dichloromethane *H. stocksii* extract. the superscripts a, b, c, and d represent significant difference (*p* < 0.05).

**Table 5:** Antibacterial activity of *Haloxylon stocksii* extracts by disc difusion method.

Strain Name	ZI of Std	ZI of AMHS	ZI of ADHS	ZI of RMHS	ZI of RDHS
*Bacillus subtilis*	23 ± 1.55	20 ± 1.33	18 ± 0.71	17 ± 1.39	19 ± 1.62
*Bacillus pumilus*	25 ± 0.77	22 ± 1.51	16 ± 1.66	15 ± 0.57	14 ± 1.26
*Micrococcus luteus*	23 ± 0.95	16 ± 0.65	19 ± 1.51	14 ± 1.19	14 ± 0.87
*Staphylococcus epidermidis*	24 ± 1.13	18 ± 0.83	17 ± 1.15	17 ± 1.28	16 ± 0.63
*Escherichia coli*	22 ± 0.47	17 ± 1.20	16 ± 1.25	19 ± 1.42	18 ± 0.96
*Bordetella bronchispetica*	25 ± 1.31	12 ± 0.33	10 ± 0.43	15 ± 1.09	14 ± 0.75
*Pseudomonas aeruginosa*	NA	9 ± 0.35	8 ± 0.21	17 ± 1.37	14 ± 1.16

Results are expressed as mean ± standard deviation. “ZI” zone of inhibition in mm; “Std” standard co-amoxiclav; “AMHS” aerial parts methanolic extract; “ADHS” aerial parts dichloromethane extract; “RMHS” roots methanolic extract; and “RDHS” roots dichloromethane extract.

**Table 6:** Molecular docking study of *H. stocksii* against α-amylase.

Sr. No.	Ligands	Binding Affinity	Amino Acid Interactions	
			Hydrogen Bonding	Pi Alkyl
**1**	Octadecanoic acid, methyl ester	−5	Gln63	Trp58, Trp59, Tyr62, Leu162, Leu165, His101, His305
**2**	4H-Pyran-4-one, 2,3-dihydro-3,5-dihydroxy-6-methyl-	−5.1	Gly309, Asp317, Arg346	
**3**	9-Octadecenoic acid, methyl ester	−5.2	Gln63	Trp59, Tyr62, Leu162, Leu165
**4**	Eicosanoic acid, methyl ester	−5.3		Trp59, Tyr62, Ala198, Leu162, Leu165, His201
**5**	1,6-Anhydro-β-ᴅ-glucopyranose	−5.8	Asn301, Gln302, Gly304, Ile312, Arg346	**Unfavorable:** Arg267
**6**	Phthalic acid, bis(7-methyloctyl) ester	−5.9	His101, Glu233, Asp300	**Pi-sigma:** Trp59, Leu162 Pi-Alkyl: Trp58, Tyr62, His305
**7**	Di-n-octyl phthalate	−6	Asp197	**Pi-sigma:** Trp59, Ile235 Alkyl/Pi-Alkyl: Trp58, Tyr62, Leu162, Leu165, Ala198, His305
**8**	Phenol, 2,2′-methylenebis[6-(1, 1-dimethylethyl)-4-(1-methylpropyl)	−8.5	His305	**Pi-Anion:** Asp300 **Pi-sigma:** Tyr62 **Pi-Pi Stacked:** Trp58, Trp59 **Alkyl/Pi-Alkyl:** Leu165, His299
**9**	γ-Sitosterol	−8.7		**Alkyl/Pi-Alkyl:** Trp59, Leu165, Ala198
	Acarbose	−6.9	Asp197, Glu233, His305, Lys352, Asp356	Pro54, Trp58, Trp59, His101, Leu162, Ser163, Leu165, Arg195, Ala198, His299, Asp300, Trp357

**Table 7:** Molecular docking study of *H. stocksii* against α-glucosidase.

Sr. No.	Ligands	Binding Affinity	Amino Acid Interactions	
			Hydrogen Bonding	Pi Alkyl
**1**	Pentadecanoic acid, 14-methyl-, methyl ester	−5	Val380, Asp401, Gly402	Val334, Val335, Lys398, Phe397
**2**	2-Furancarboxaldehyde, 5-(hydroxymethyl)-	−5.2	His515	**Pi-Alkyl:** Lys352, Ala514 **Amide-Pi Stacked:** Phe516
**3**	Ethyl Oleate	−5.4	Arg457	**Pi-sigma:** Phe463 **Alkyl/Pi-Alkyl:** Leu95, Arg456
**4**	Eicosanoic acid, methyl ester	−5.4	Arg347, Gly432	His348, Ala349, Lys352, Ala444, Arg450, Ala454
**5**	1,6-Anhydro-β-ᴅ-glucopyranose	−5.7	Arg437, Asp441, Ala451	
**6**	Di-n-octyl phthalate	−5.6	Gly439, Gly581, His515	**Pi-sigma:** Ala349 Alkyl/Pi-Alkyl: Ala43, Leu93
**7**	Phthalic acid, bis(7-methyloctyl) ester	−6.2	Ser44, Asn443, Arg450	**Pi-sigma:** Phe516 Alkyl/Pi-Alkyl: Tyr41, Lys352, Val435
**8**	Phenol, 2,2′-methylenebis[6-(1, 1-dimethylethyl)-4-(1-methylpropyl)	−8.1	Asp441	**Pi-Cation:** His348 Alkyl/Pi-Alkyl: Pro442, Ala444, Arg450, Ala451
**9**	γ-Sitosterol	−8.9	Arg437	His348, Ala349, Lys352, Ala444, Ala451, Ala454
	Acarbose	−6.8	Arg17, Asp59, Asn61, Asp381, Pro433, Trp434	Asn58, Asp60, Asp378, Asp379, Asp382, Val383, Met435

**Table 8:** Pharmacokinetic properties of best-docked compounds.

Sr. No.	Compound Name	Gastrointestinal Absorption	Blood–brain Barrier Permeant	Pgp Inhibitor	CYP1A2 Inhibitor	CYP2C19 Inhibitor	CYP2C9 Inhibitor	CYP2D6 Inhibitor	CYP3A4 Inhibitor	Log Kp (cm/s)
**1.**	Octadecanoic acid, methyl ester	High	No	No	Yes	No	No	No	No	−2.19
**2.**	4H-Pyran-4-one, 2,3-dihydro-3,5-dihydroxy-6- methyl-	High	No	No	No	No	No	No	No	−7.44
**3.**	Ethyl Oleate	High	No	No	Yes	No	No	No	No	−2.82
**4.**	Eicosanoic acid, methyl ester	Low	No	No	Yes	No	No	No	No	−1.69
**5.**	1,6-Anhydro-β-ᴅ-glucopyranose	High	No	Yes	No	No	No	No	No	−8.82
**6.**	Phthalic acid, bis(7-methyloctyl) ester	Low	No	No	No	No	No	No	Yes	−3.61
**7.**	Di-n-octyl phthalate	High	No	No	No	No	No	No	Yes	−2.93
**8.**	Phenol,2,2′-methylenebis[6-(1,1-dimethylethyl)-4-(1- methylpropyl)	Low	No	Yes	No	No	No	Yes	No	−2.60
**9.**	γ-Sitosterol	Low	No	No	No	No	No	No	No	−2.20
**10.**	Acarbose	Low	No	Yes	No	No	No	No	No	−16.29

**Table 9:** Lipinski rule of five and solubility of best-docked compounds.

Sr. No.	Compounds Name	Lipinski’s rule						Solubility		
		HBD	HBA	MWT (g/moL)	Lipophilicity	MR	LR	ESOL Class	Ali Class	Silicos-IT Class
1.	Octadecanoic acid, methyl ester	0	2	298.50	4.81	94.73	1	Moderately soluble	Poorly soluble	Poorly soluble
2.	4H-Pyran-4-one, 2,3- dihydro-3,5-dihydroxy-6- methyl-	2	4	144.13	1.19	32.39	0	Very soluble	Very soluble	Soluble
3.	Ethyl Oleate	0	2	296.49	4.75	94.26	1	Moderately soluble	Poorly soluble	Poorly soluble
4.	Eicosanoic acid, methyl ester	0	2	326.56	5.35	104.35	1	Poorly soluble	Poorly soluble	Poorly soluble
5.	1,6-Anhydro-β-ᴅ-glucopyranose	3	5	162.14	1.27	32.38	0	Highly soluble	Highly soluble	Soluble
6.	Phthalic acid, bis(7- methyloctyl) ester	0	4	418.61	5.41	125.91	1	Poorly soluble	Poorly soluble	Poorly soluble
7.	Di-n-octyl phthalate	0	4	390.56	4.14	116.30	1	Poorly soluble	Poorly soluble	Poorly soluble
8.	Phenol, 2,2′-methylenebis[6-(1,1-dimethylethyl)-4-(1- methylpropyl)	2	2	424.66	4.95	137.25	1	Poorly soluble	Poorly soluble	Poorly soluble
9.	γ-Sitosterol	1	1	414.71	4.79	133.23	1	Poorly soluble	Poorly soluble	Poorly soluble
10.	Acarbose	14	19	645.60	0.63	136.69	3	Highly soluble	Highly soluble	Soluble

HBD: hydrogen bond donor; HBA: hydrogen bond acceptor; MWT: molecular weight; MR: molar refractivity; LR: Lipinski rule.

**Table 10:** Toxicity profiles of best-docked molecules.

Sr. No.	Compound Name	Predicted LD_50_ (mg/kg)	Predicted Toxicity Class	Hepatotoxicity	Carcinogenicity	Immunotoxicity	Mutagenicity	Cytotoxicity
1.	Octadecanoic acid, methyl ester	5000	5	Not active	Not active	Not active	Not active	Not active
2.	4H-Pyran-4-one, 2,3- dihydro-3,5-dihydroxy-6- methyl-	595	4	Not active	Not active	Not active	Active	Not active
3.	Ethyl oleate	3000	5	Not active	Not active	Not active	Not active	Not active
4.	Eicosanoic acid, methyl ester	5000	5	Not active	Not active	Not active	Not active	Not active
5.	1,6-Anhydro-β-ᴅ-glucopyranose	23,000	6	Not active	Not active	Not active	Not active	Not active
6.	Phthalic acid, bis(7- methyloctyl) ester	1340	4	Not active	Active	Inactive	Not active	Not active
7.	Di-n-octyl phthalate	1340	4	Not active	Active	Not active	Not active	Not active
8.	Phenol, 2,2′-methylenebis[6- (1, 1-dimethylethyl)-4-(1- methylpropyl)	3430	5	Not active	Not active	Not active	Not active	Not active
9.	γ-Sitosterol	890	4	Not active	Not active	Active	Not active	Not active
10.	Acarbose	24,000	6	Active	Not active	Active	Not active	Not active

## Data Availability

Not applicable.

## Supplementary Materials

The following supporting information can be downloaded at: https://www.mdpi.com/article/10.3390/molecules28093847/s1, Figure S1: Retention time peak, fragmentation pattern and chemical structure of N-Methyltyramine; Figure S2: Retention time peak, fragmentation pattern and chemical structure of Acetophenone; Figure S3: Retention time peak, fragmentation pattern and chemical structure of Hordenine; Figure S4: Retention time peak, fragmentation pattern and chemical structure of Ethyl 2-(3-{[4-(4-methylphenyl)-1,3-thiazol-2-yl]methyl}-4-oxo-3,4-dihydrophthalazin-1-yl)acetate; Figure S5: Retention time peak, fragmentation pattern and chemical structure of Fraxetin; Figure S6: Retention time peak, fragmentation pattern and chemical structure of 6,8-dihydroxy-7-methoxy-3-methyl-1H-isochromen-1-one; Figure S7: Retention time peak, and fragmentation pattern of Unknown compound (not identified with library); Figure S8: Retention time peak, fragmentation pattern and chemical structure of nor -3-methylfentanyl; Figure S9: Retention time peak, fragmentation pattern and chemical structure of Moupinamide; Figure S10: Retention time peak, fragmentation pattern and chemical structure of 2-(2,6-dimethoxyphenyl)-5,6-dimethoxy-4H-chromen-4-one; Figure S11: Retention time peak, fragmentation pattern and chemical structure of Piperine: Figure S12: Retention time peak, fragmentation pattern and chemical structure of tris(2-Butoxyethyl) phosphate; Figure S13: Retention time peak, fragmentation pattern and chemical structure of Acetyl tributyl citrate; Figure S14: Retention time peak, and fragmentation pattern of Unknown compound (not identified with library); Figure S15: Retention time peak, and fragmentation pattern of Unknown compound (not identified with library); Figure S16: Retention time peak, fragmentation pattern and chemical structure of Hexadecanamide; Figure S17: Retention time peak, fragmentation pattern and chemical structure of Unknown compound (not identified with library); Figure S18: Retention time peak, fragmentation pattern and chemical structure of 1-Palmitoylglycerol; Figure S19: Retention time peak, fragmentation pattern and chemical structure of (1S)-Tricyclo[7.3.1.0~2,7~]tridec-2(7)- en-13-one; Figure S20: Retention time peak, fragmentation pattern and chemical structure of Stearamide; Figure S21: Retention time peak, fragmentation pattern and chemical structure of Erucamide; Figure S22: Retention time peak, fragmentation pattern and chemical structure of unknown compound (not identified with library); Figure S23: Retention time peak, fragmentation pattern and chemical structure of N,N-Dimethylaniline. Table S1: Safety Profile of LC-MS identified compound; Table S2: α-amylase inhibition and α-glucosidase inhibition of *Haloxylon stocksii*.

## Sample Availability

Samples of the compounds are not available from the authors.
