# Supplementary material for: Chemical Characterisation, Antidiabetic, Antibacterial, and In Silico Studies for Different Extracts of Haloxylon stocksii (Boiss.) Benth: A Promising Halophyte
Source: Molecules. 2023 May 1;28(9):3847. doi: 10.3390/molecules28093847 (PMC10180423; doi:10.3390/molecules28093847)
Supplement: Supplementary file 1 [file molecules-28-03847-s001.zip › molecules-2335960-supplementary.pdf]

molecules

Supplementary file

Chemical Characterisation, Antidiabetic, Antibacterial, and In silico Studies for Different Extracts of *Haloxylon stocksii* (Boiss.) Benth: A Promising Halophyte

Syed Nabil Raza Rizvi [1], Samina Afzal [1,*], Kashif-ur-Rehman Khan [2,*], Hanan Y. Aati [3], Huma Rao [2], Bilal Ahmad Ghalloo [2,4], Muhammad Nadeem Shahzad [2], Duraiz Ahmed Khan [2], Tuba Esatbeyoglu [5,*] and Sameh A. Korma [6,7]

[1] Department of Pharmaceutical Chemistry, Faculty of Pharmacy, Bahauddin Zakariya University, Multan 60000, Pakistan
[2] Department of Pharmaceutical Chemistry, Faculty of Pharmacy, The Islamia University of Bahawalpur, Bahawalpur 63100, Pakistan;
[3] Department of Pharmacognosy, College of Pharmacy, King Saud University, Riyadh 11495, Saudi Arabia
[4] Department of Medicinal Chemistry, College of Pharmacy, University of Minnesota, Minneapolis, MN 55454, USA
[5] Department of Food Development and Food Quality, Institute of Food Science and Human Nutrition, Gottfried Wilhelm Leibniz University Hannover, Am Kleinen Felde 30, 30167 Hannover, Germany
[6] Department of Food Science, Faculty of Agriculture, Zagazig University, Zagazig 44519, Egypt
[7] School of Food Science and Engineering, South China University of Technology, Guangzhou 510641, China
* Correspondence: samina.afzal@bzu.edu.pk (S.A.); esatbeyoglu@lw.uni-hannover.de (T.E.)

Table S1: Safety Profile of LC-MS identified compound

Compound Name	Toxicity profile
N-Methyltyramine	LD_{50} = 780 mg/kg (Intraperitoneal)<br>LD_{50} = 275 mg/kg (Intravenous)
Acetophenone	Safe, Hypnosis occur at 0.4 to 0.5 g/kg (Intravenous)
Hordenine	Harmful if swallowed, skin sensitization
Fraxetin	NA
6,8-dihydroxy-7-methoxy-3-methyl-1H-isochromen-1-one	NA
Nor-3-methylfentanyl	NA
Moupinamide	Very toxic to aquatic life
2-(2,6-dimethoxyphenyl)-5,6-dimethoxy-4H-chromen-4-one	NA
Piperine	LD_{50} = 34 mg/kg (Rats, Intraperitoneal)<br>LD_{50} = 71 mg/kg (Mice, Intravenous)
Tris(2-butoxyethyl) phosphate	No skin sensitization
Acetyl tributyl citrate	No skin sensitization
Hexadecanamide	May cause irritation
1-Palmitoylglycerol	May cause irritation
(1S)-Tricyclo[7.3.1.0~2,7~]tridec-2(7)- en-13-one	NA
Stearamide	Cough and redness to the skin
Erucamide	Myocardial granulomas, necrosis, and lesions
N,N-Dimethylaniline	LD_{50} = 50 mg/kg (Oral)

"NA" No toxicity data is available in https://pubchem.ncbi.nlm.nih.gov/ database.

Table S2: α-amylase inhibition and α-glucosidase inhibition **of** *Haloxylon stocksii*

Sample Name	α-amylase inhibition	α-glucosidase inhibition
**AMHS**	65.78±3.19^{b}	59.91±4.16^{b}
**ADHS**	42.64±1.62^{d}	38.03±2.25^{d}
**RMHS**	58.82±2.92^{c}	53.41±5.64^{c}
**RDHS**	44.47±1.22^{d}	32.50±0.85^{e}
**Acarbose**	79.58±4.28^{a}	74.27±6.66^{a}

"AMHS" aerial parts methanolic *H. stocksii* extract, "ADHS" Aerial parts dichloromethane *H. stocksii* extract, "RMHS" roots methanolic *H. stocksii* extract, and "RDHS" roots dichloromethane *H. stocksii* extract; the superscripts a, b, c, d, and e represent significant difference (p<0.05)

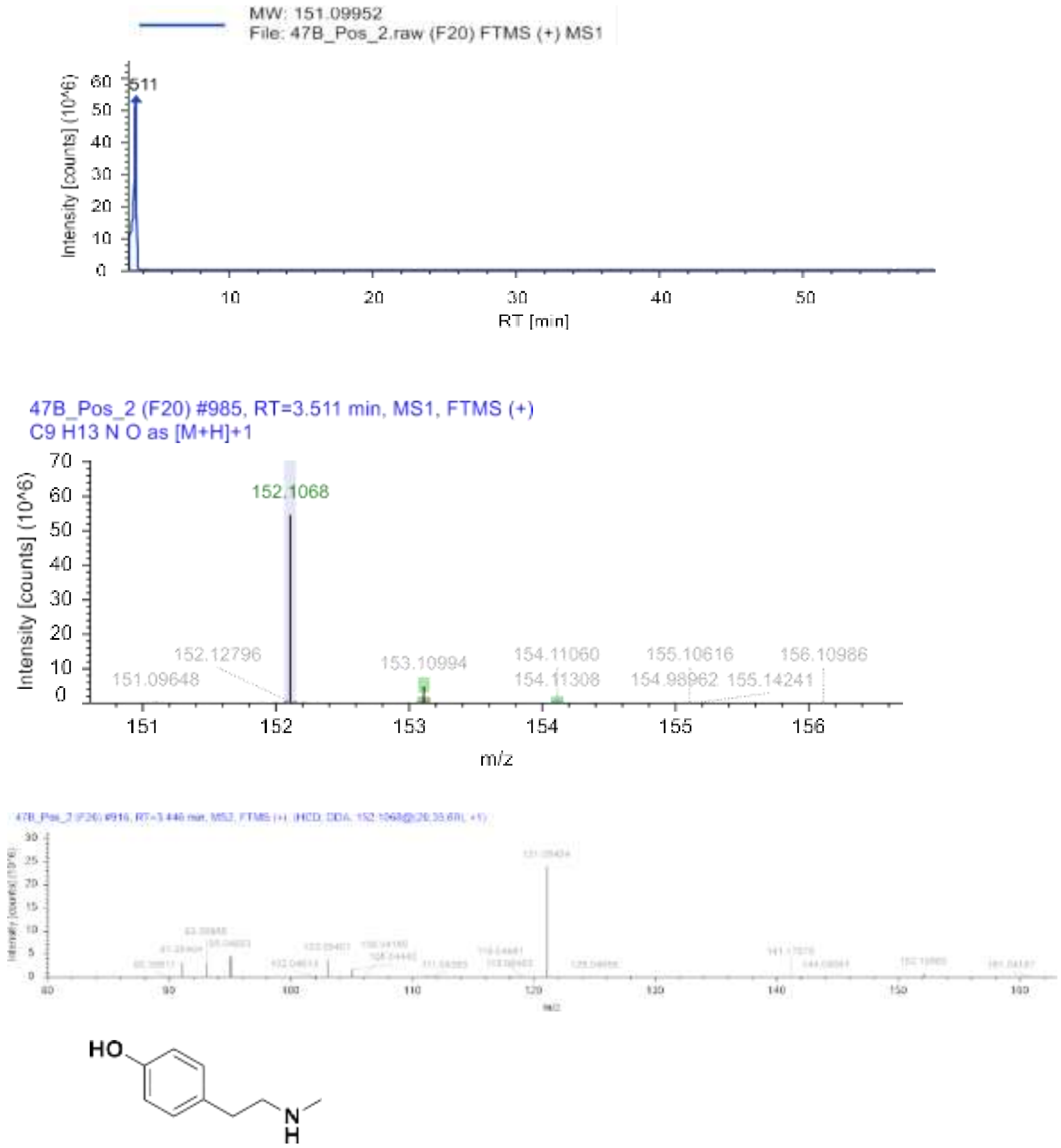

Figure S1: Retention time peak, fragmentation pattern and chemical structure of N-Methyltyramine

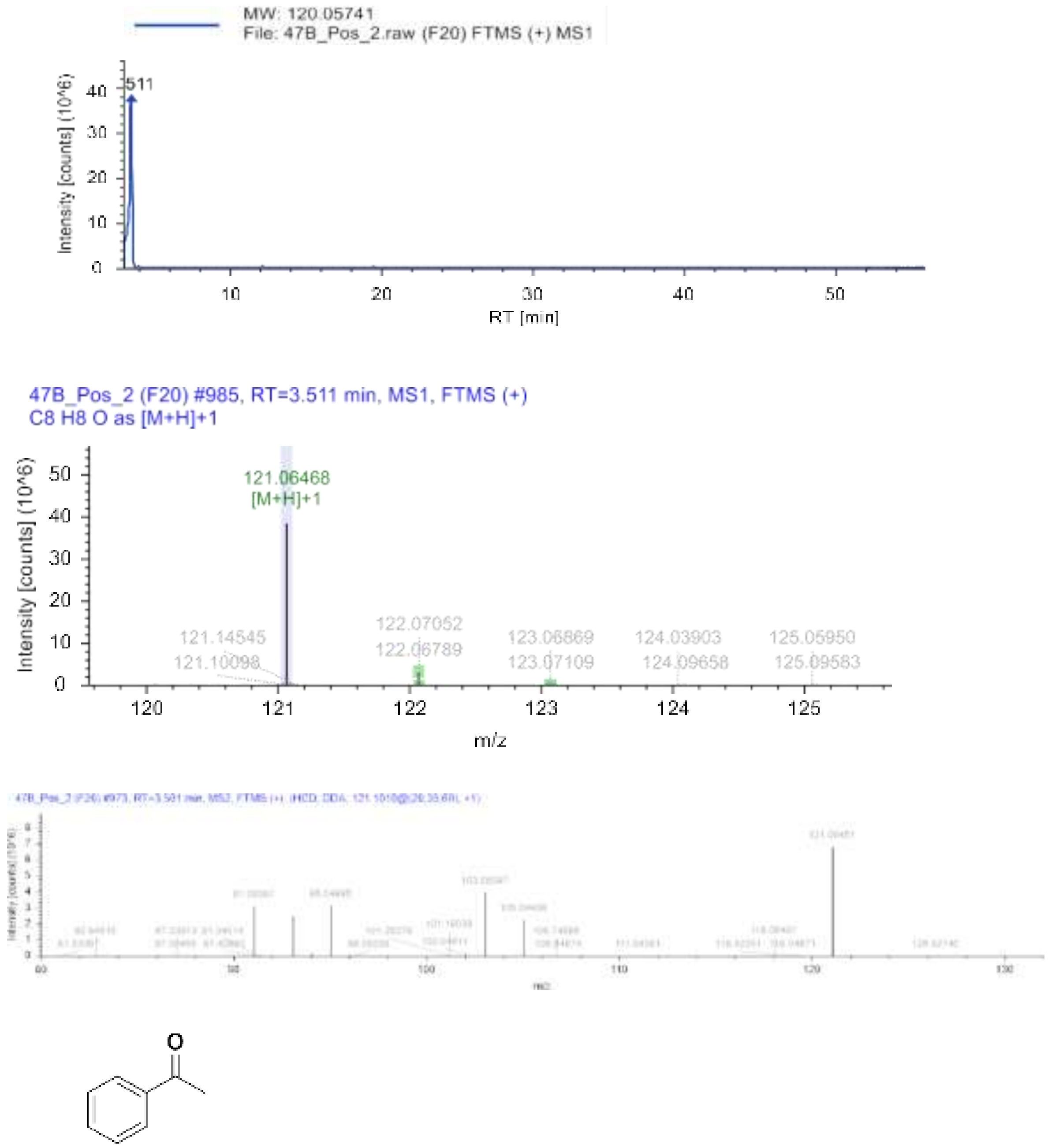

Figure S2: Retention time peak, fragmentation pattern and chemical structure of Acetophenone

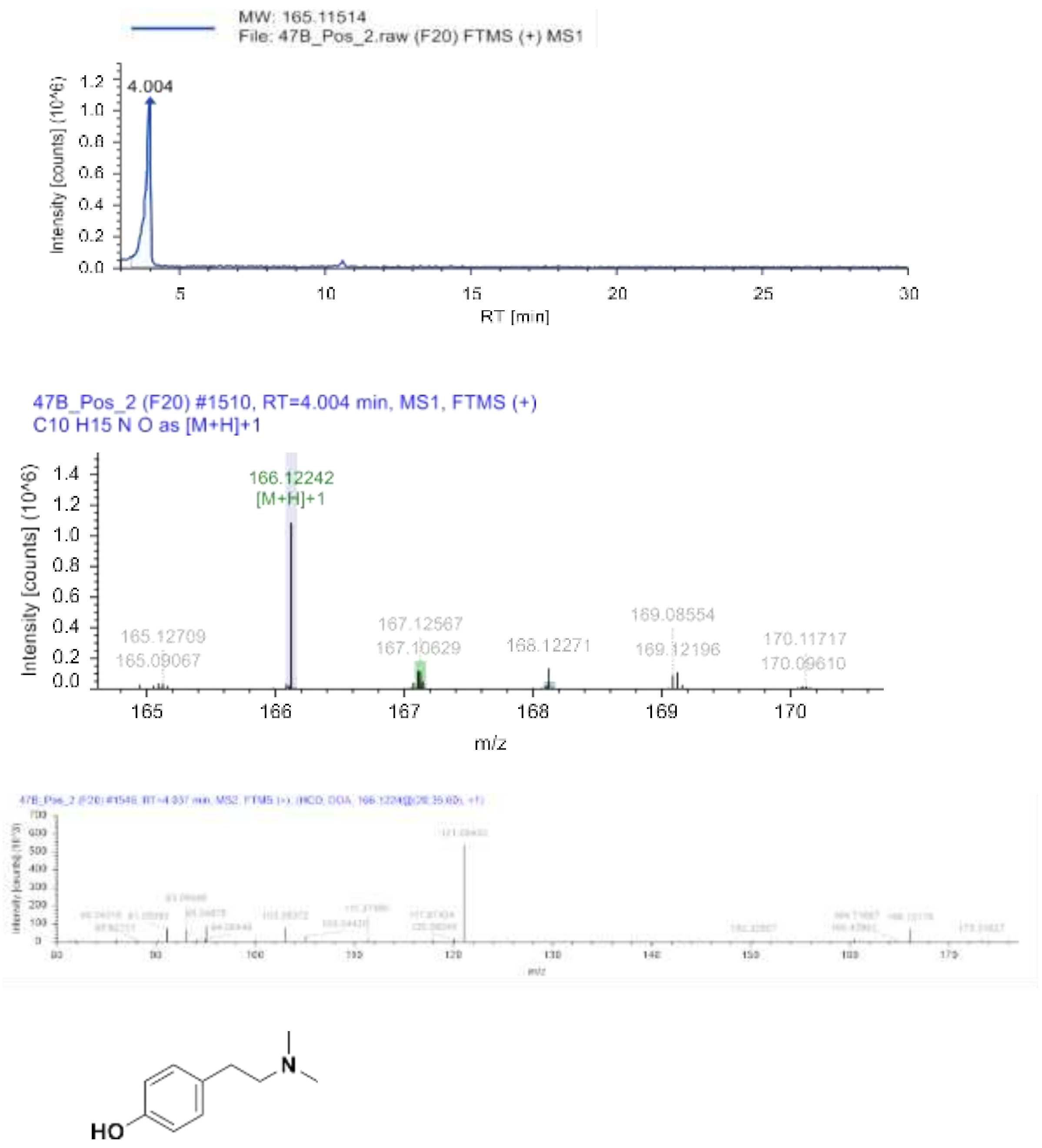

Figure S3: Retention time peak, fragmentation pattern and chemical structure of Hordenine

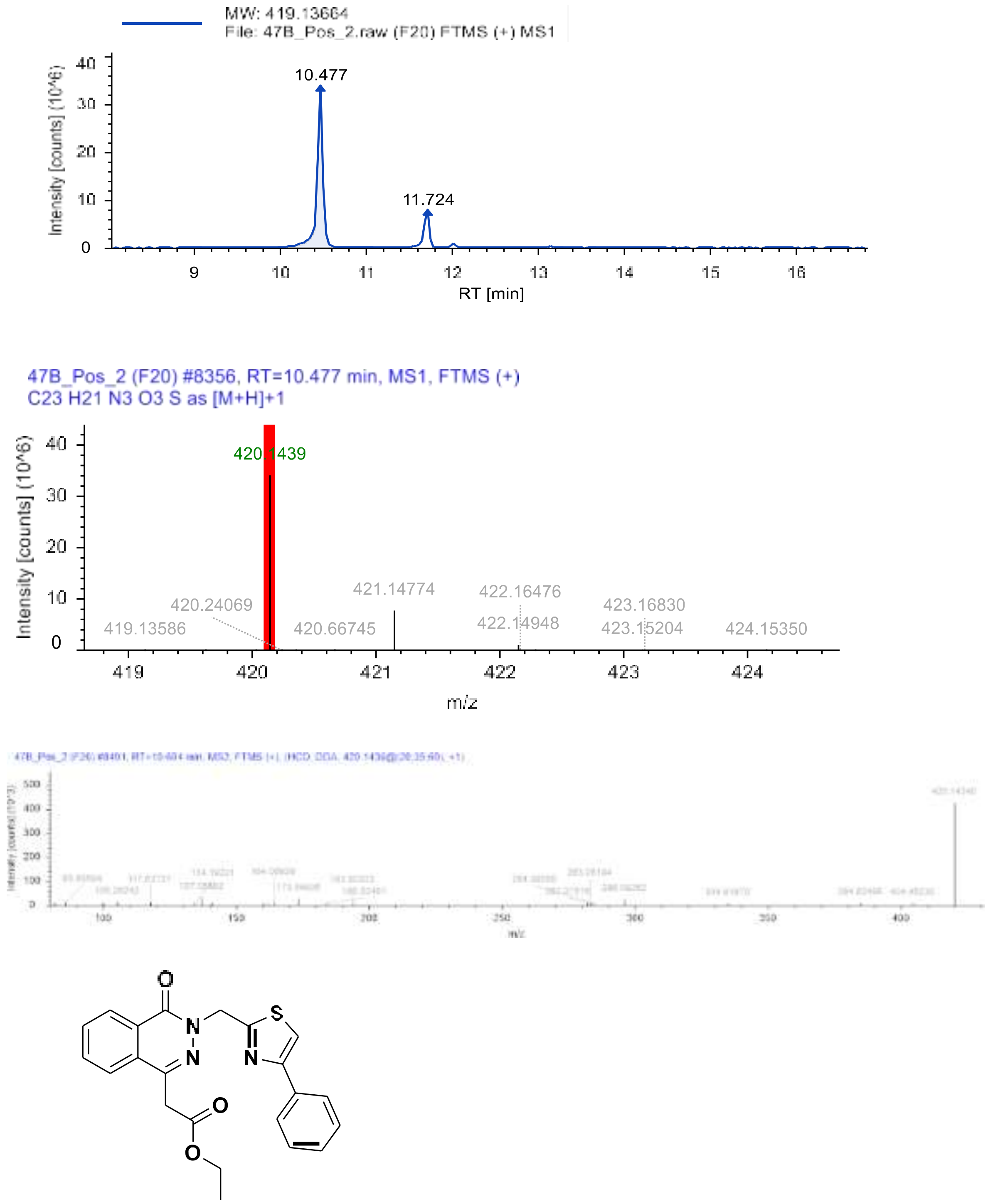

Figure S4: Retention time peak, fragmentation pattern and chemical structure of Ethyl 2-(3-{[4-(4-methylphenyl)-1,3-thiazol-2-yl]methyl}-4-oxo-3,4-dihydrophthalazin-1-yl)acetate

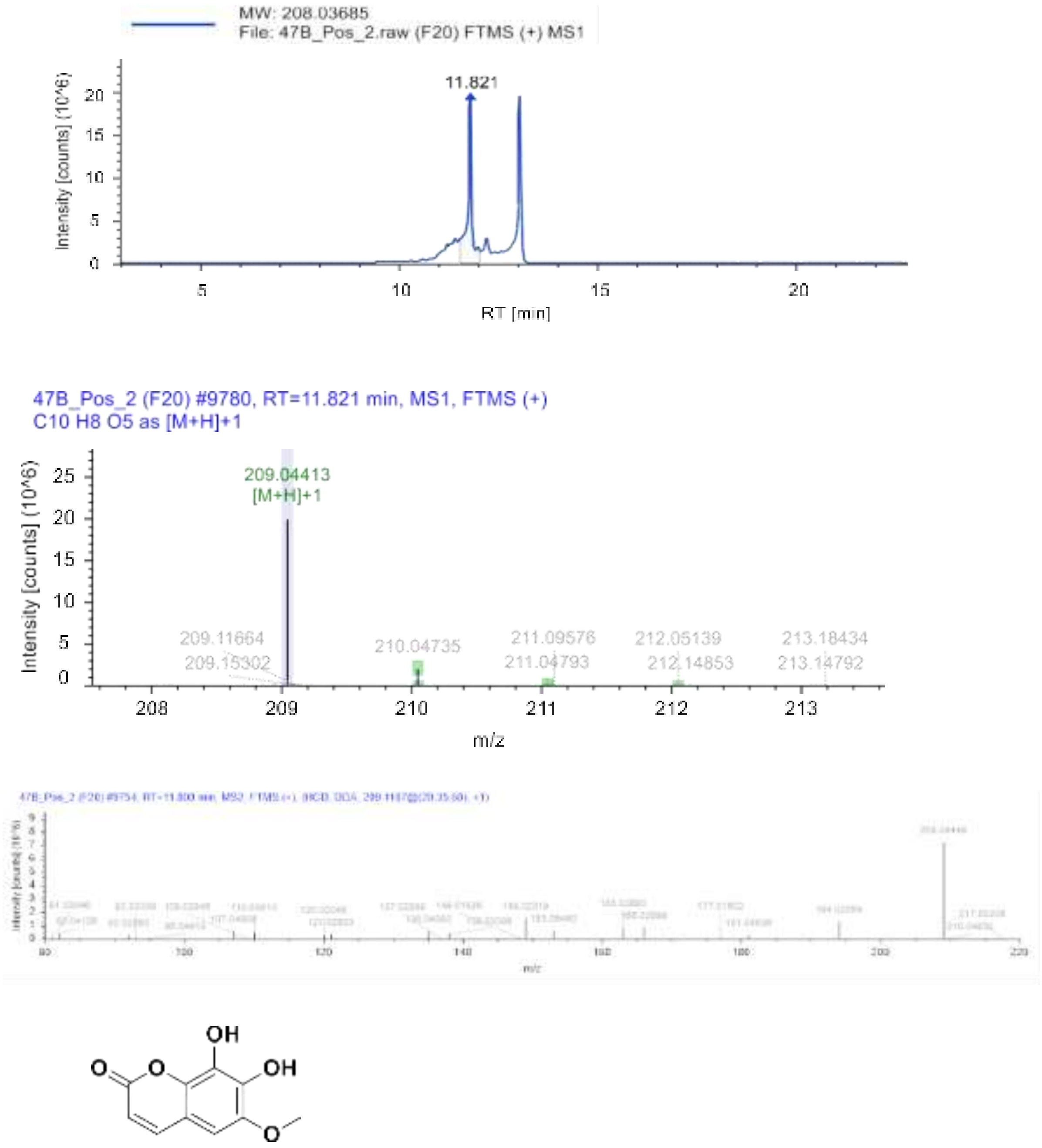

Figure S5: Retention time peak, fragmentation pattern and chemical structure of Fraxetin

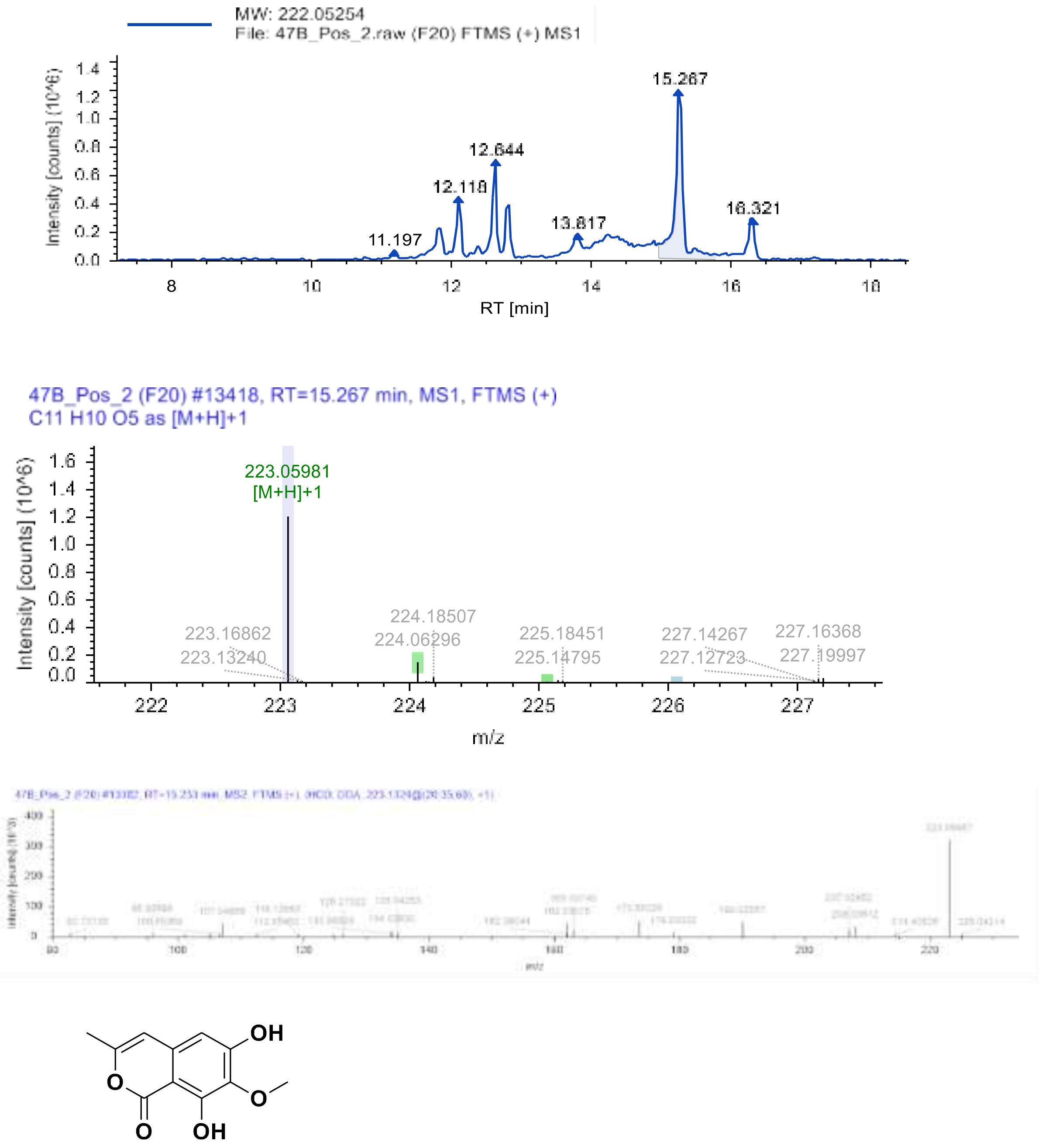

Figure S6: Retention time peak, fragmentation pattern and chemical structure of 6,8-dihydroxy-7-methoxy-3-methyl-1H-isochromen-1-one

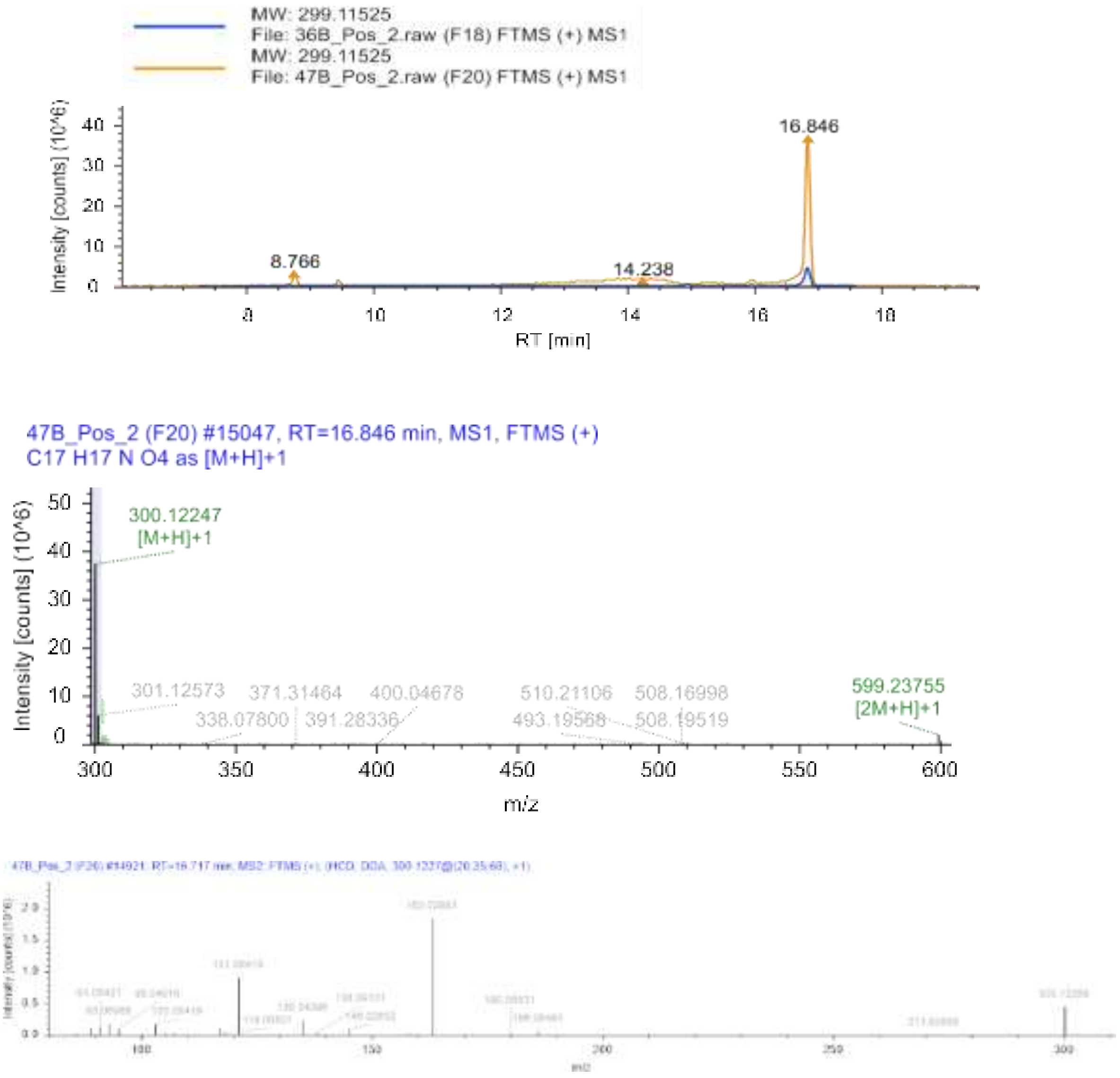

Figure S7: Retention time peak, and fragmentation pattern of Unknown compound (not identified with library)

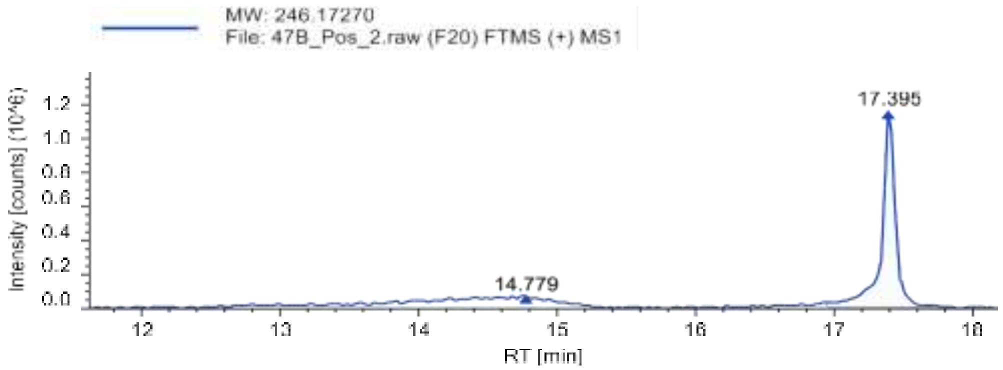

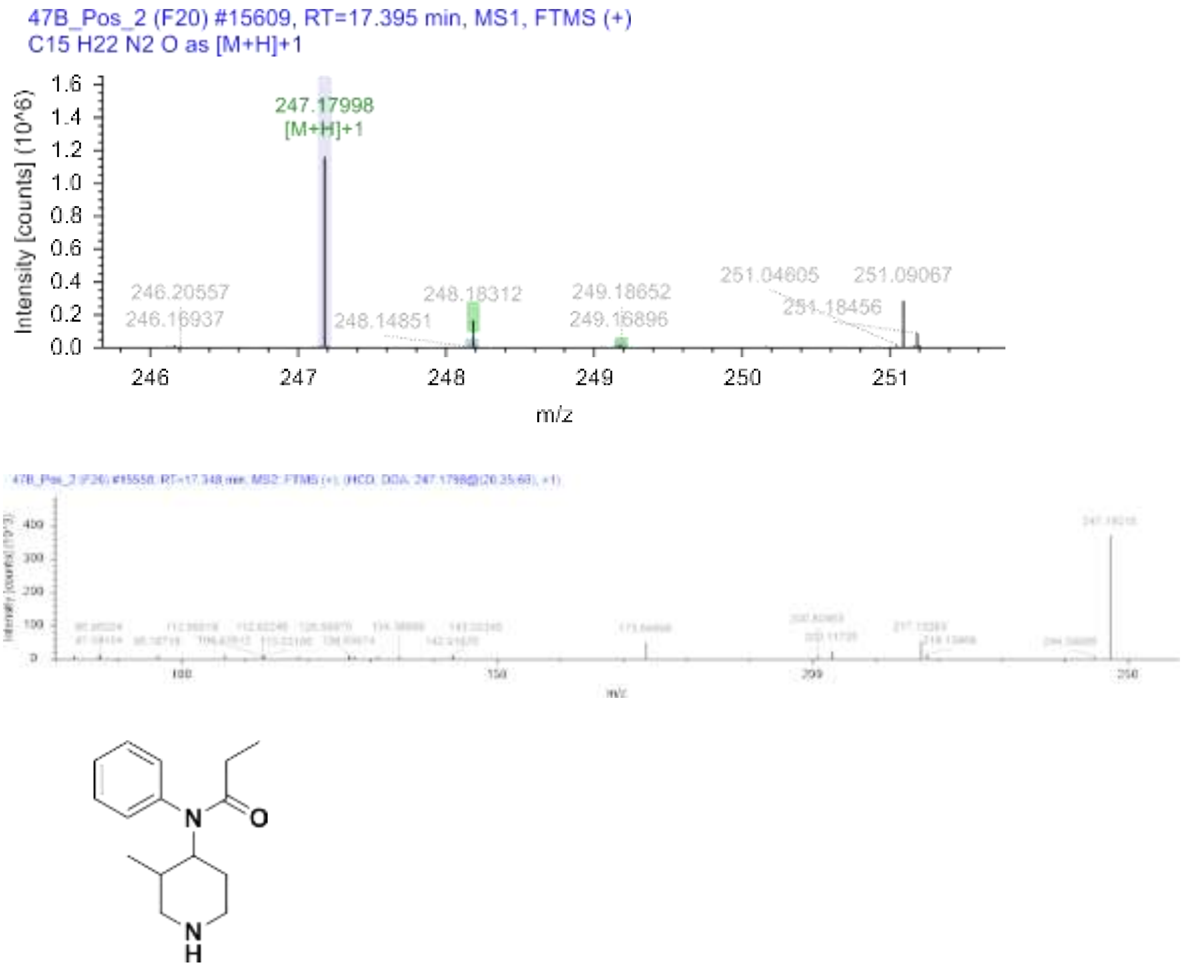

Figure S8: Retention time peak, fragmentation pattern and chemical structure of nor -3-methylfentanyl

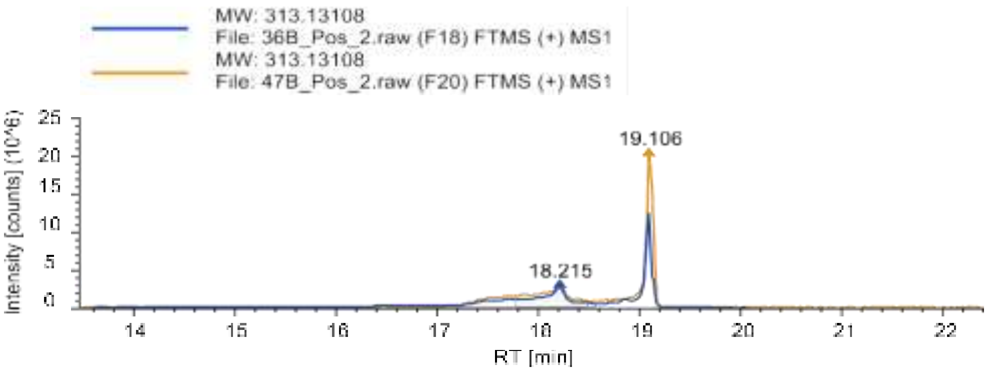

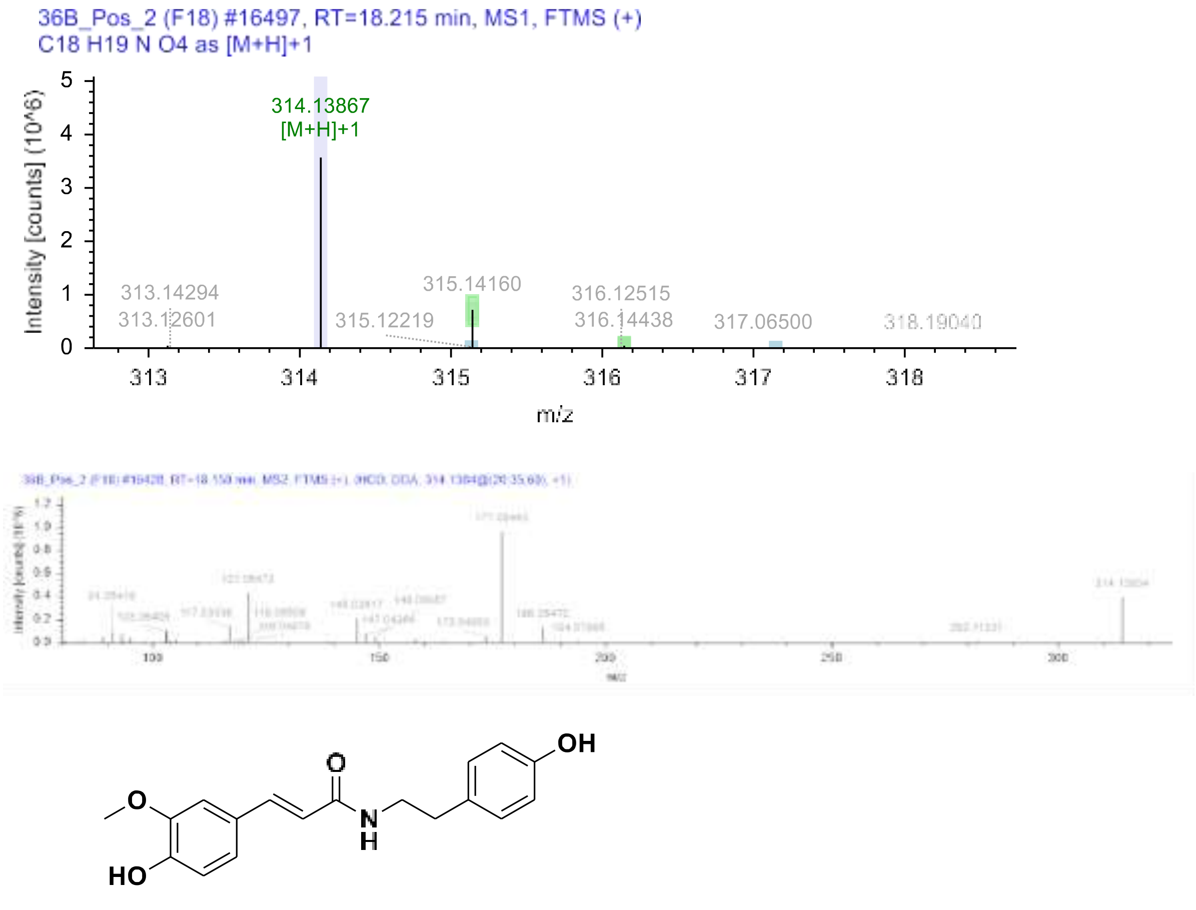

Figure S9: Retention time peak, fragmentation pattern and chemical structure of Moupinamide

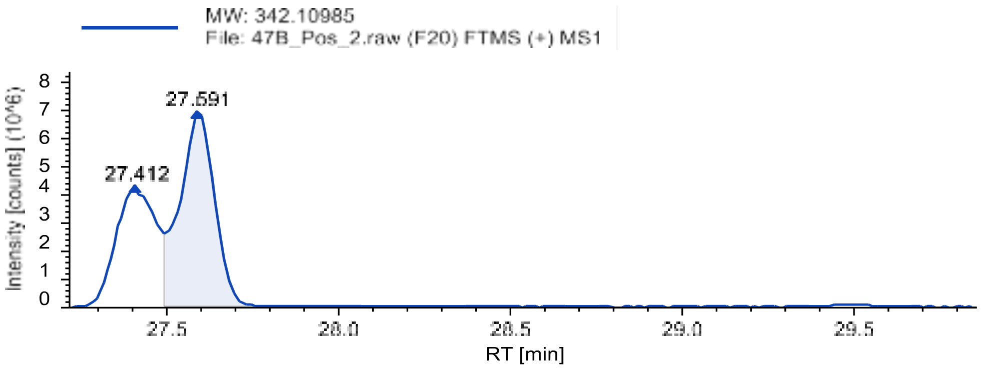

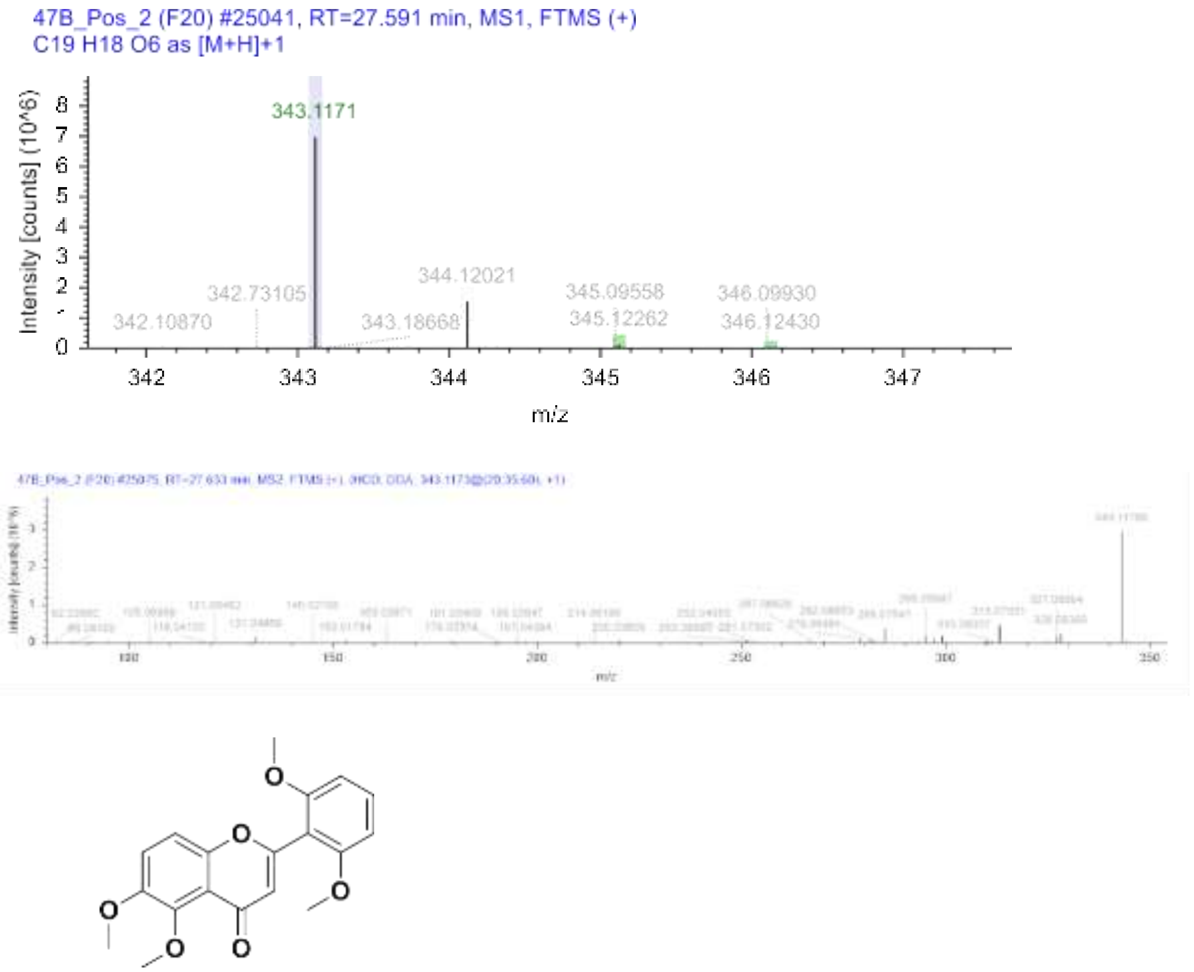

Figure S10: Retention time peak, fragmentation pattern and chemical structure of 2-(2,6-dimethoxyphenyl)-5,6-dimethoxy-4H-chromen-4-one

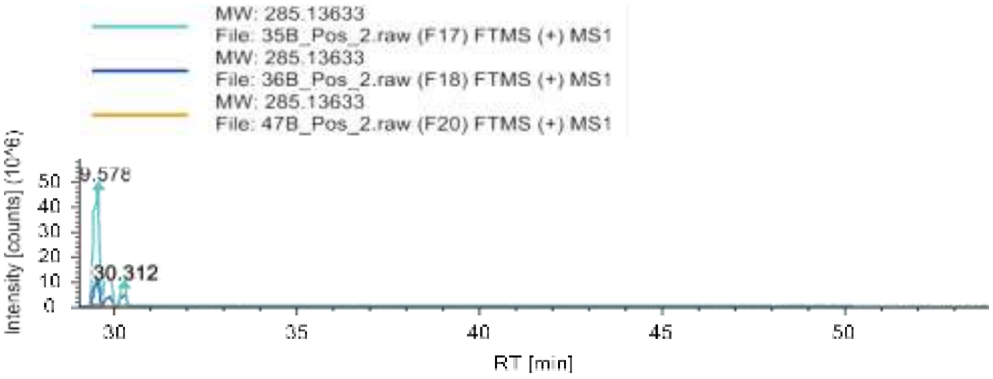

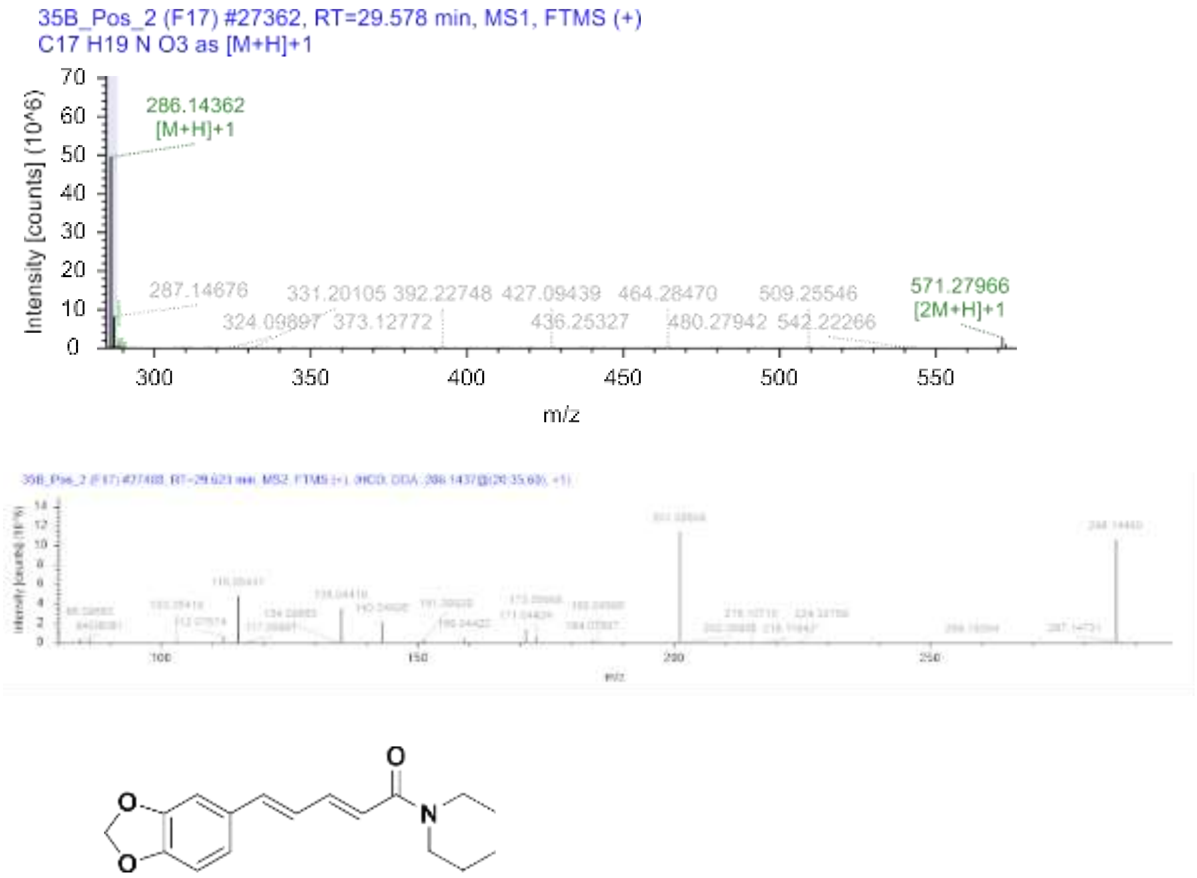

Figure S11: Retention time peak, fragmentation pattern and chemical structure of Piperine

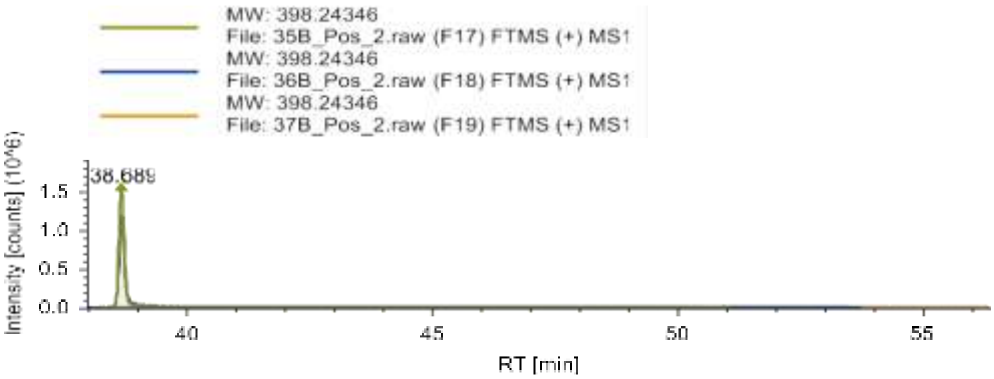

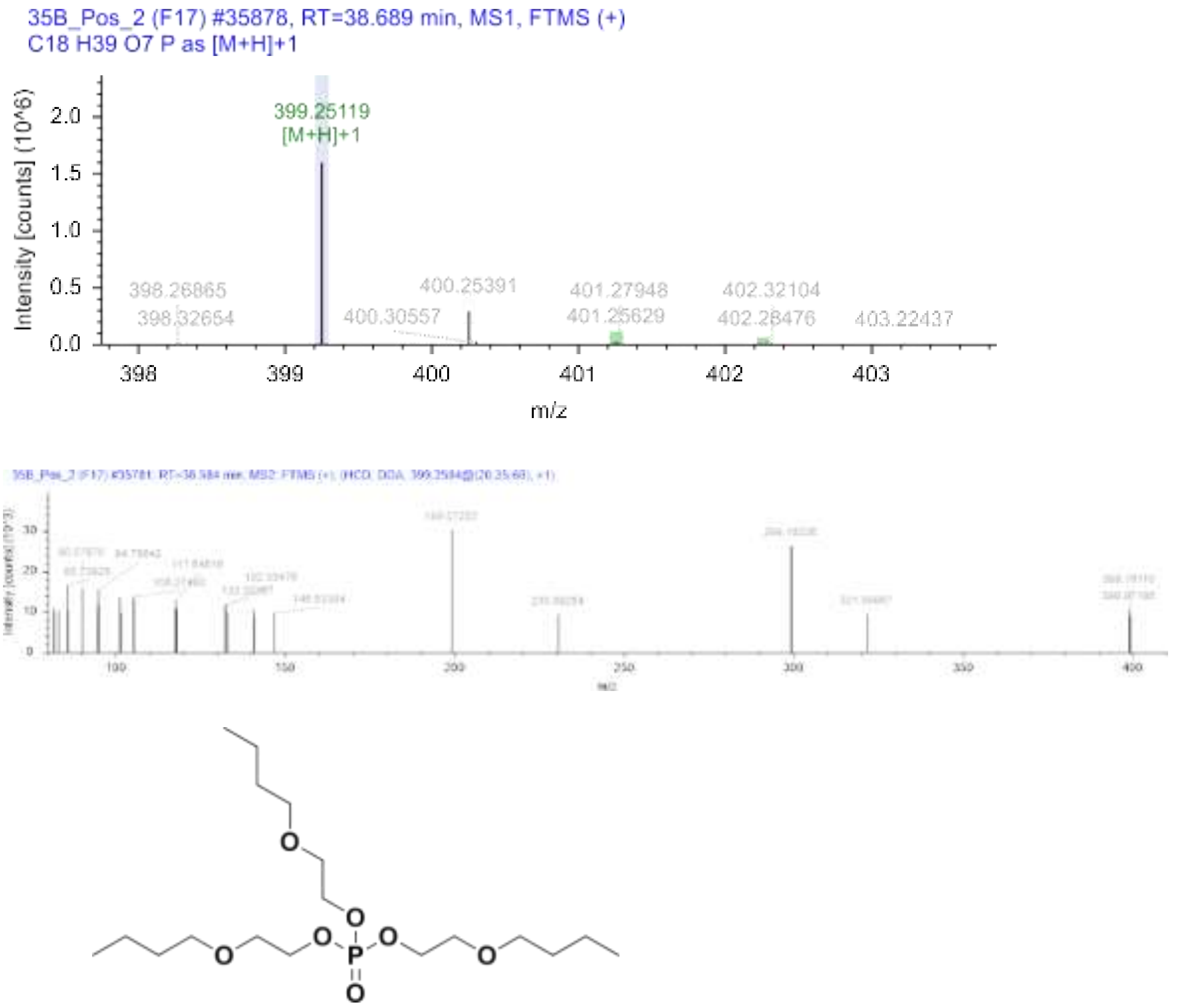

Figure S12: Retention time peak, fragmentation pattern and chemical structure of tris(2-Butoxyethyl) phosphate

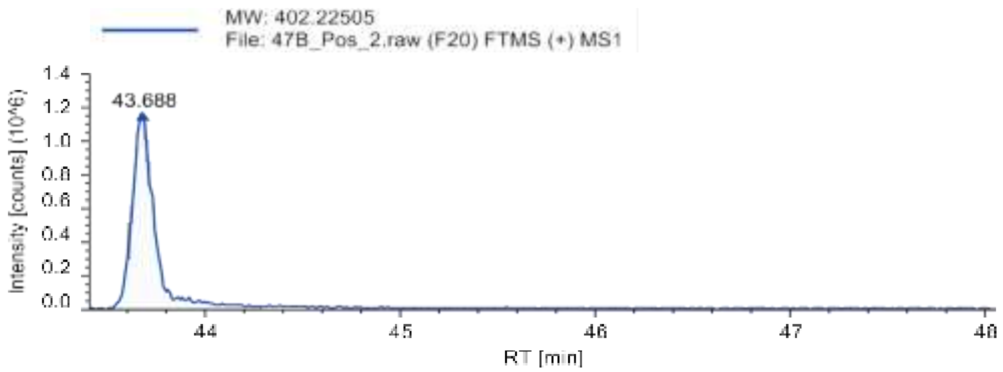

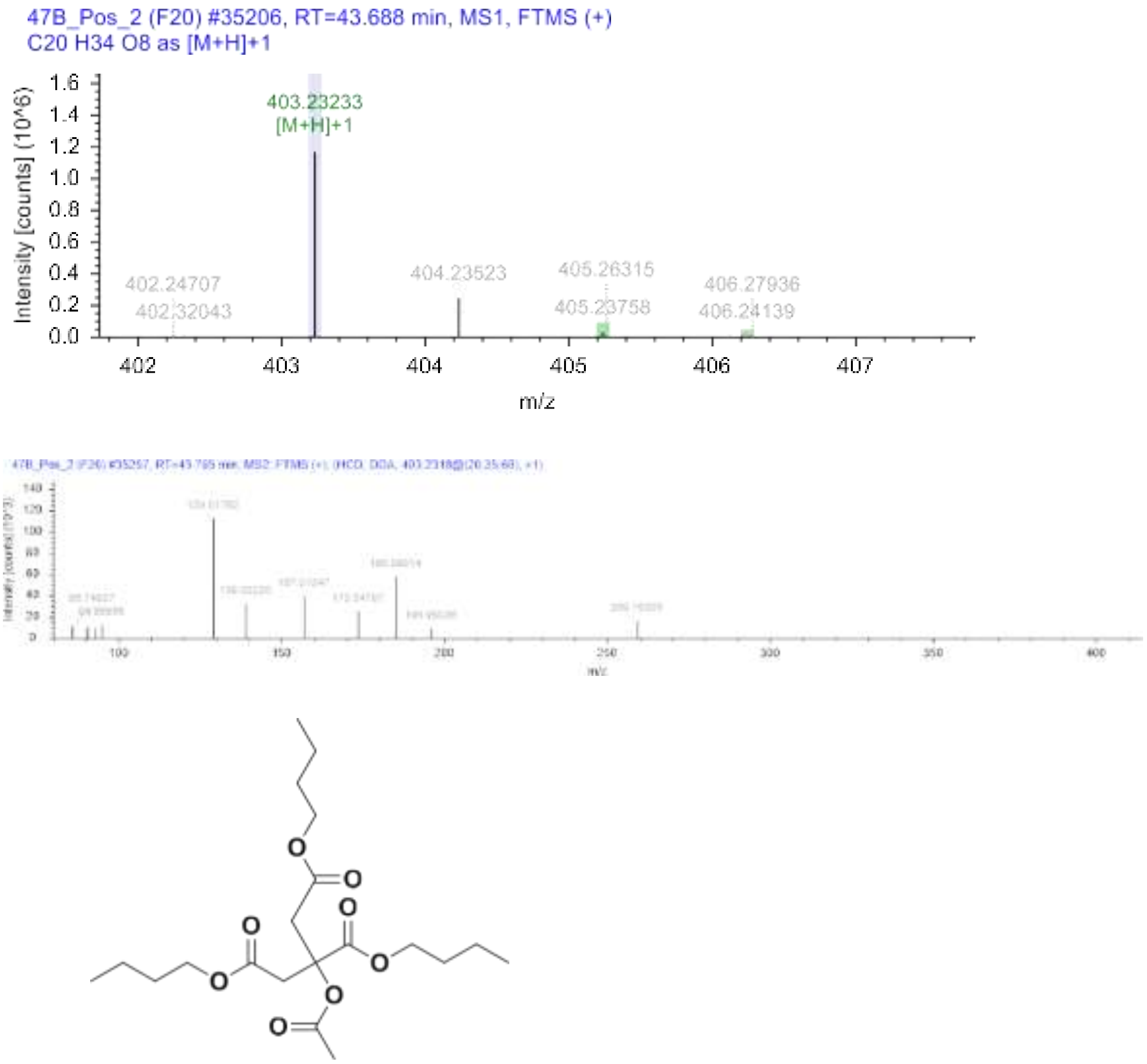

Figure S13: Retention time peak, fragmentation pattern and chemical structure of Acetyl tributyl citrate

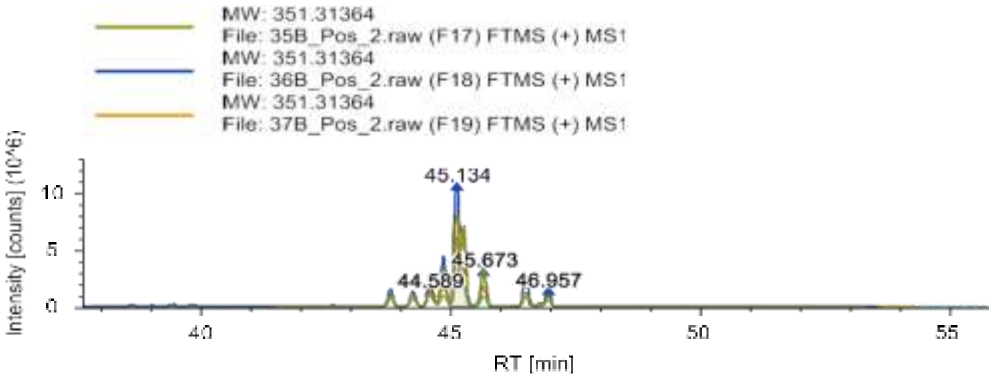

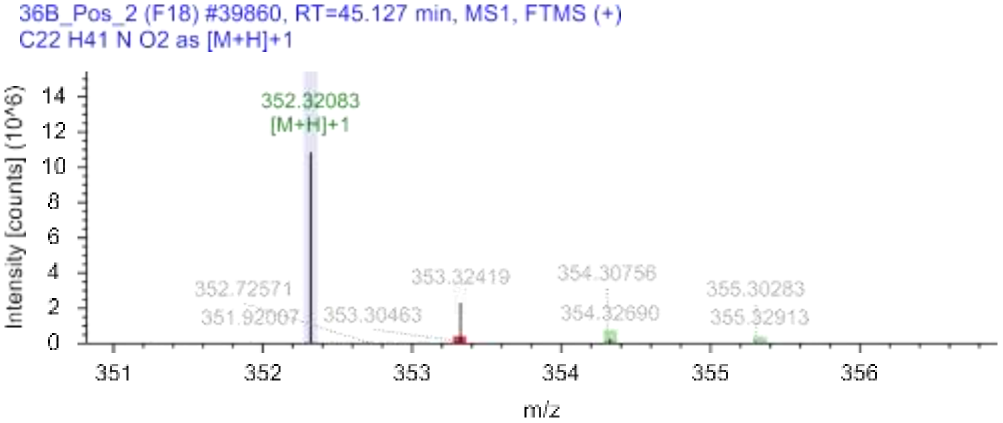

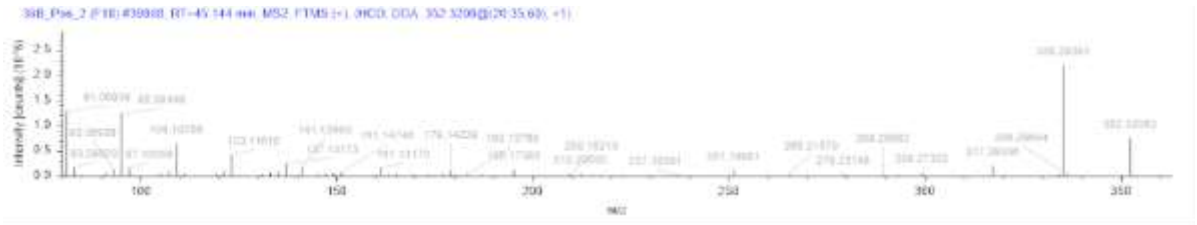

Figure S14: Retention time peak, and fragmentation pattern of Unknown compound (not identified with library)

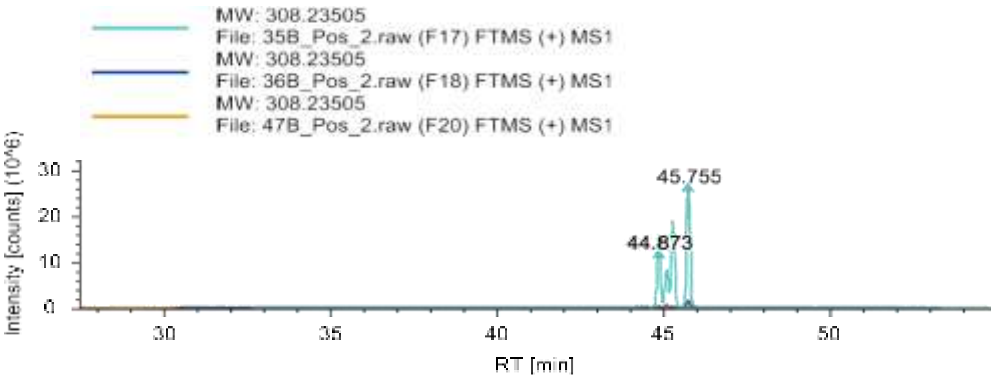

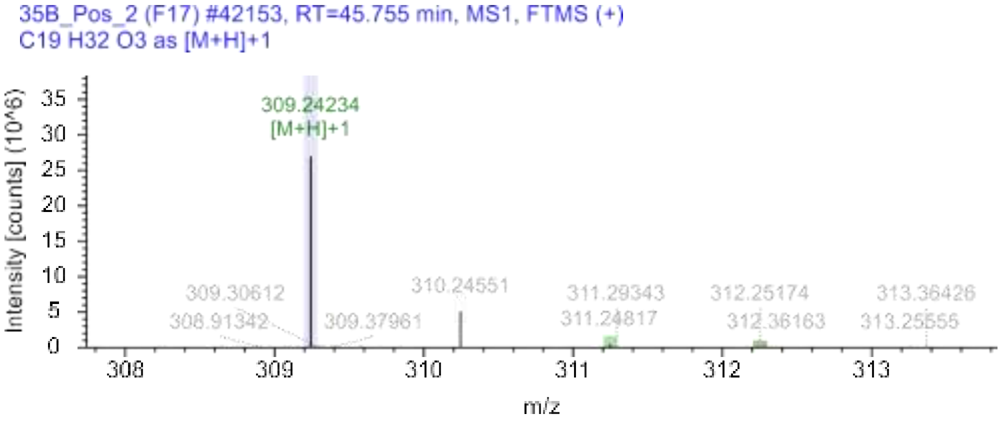

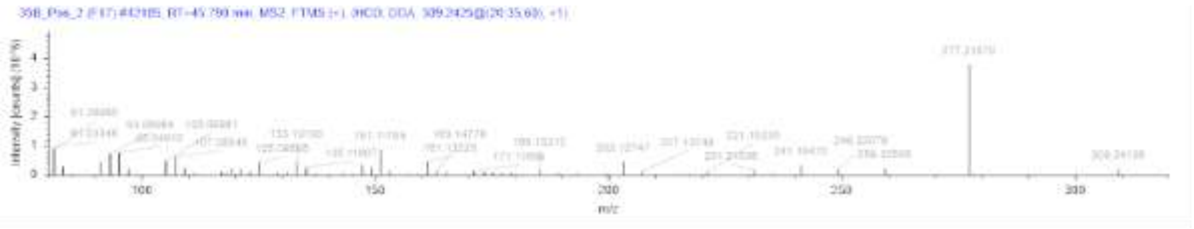

Figure S15: Retention time peak, and fragmentation pattern of Unknown compound (not identified with library)

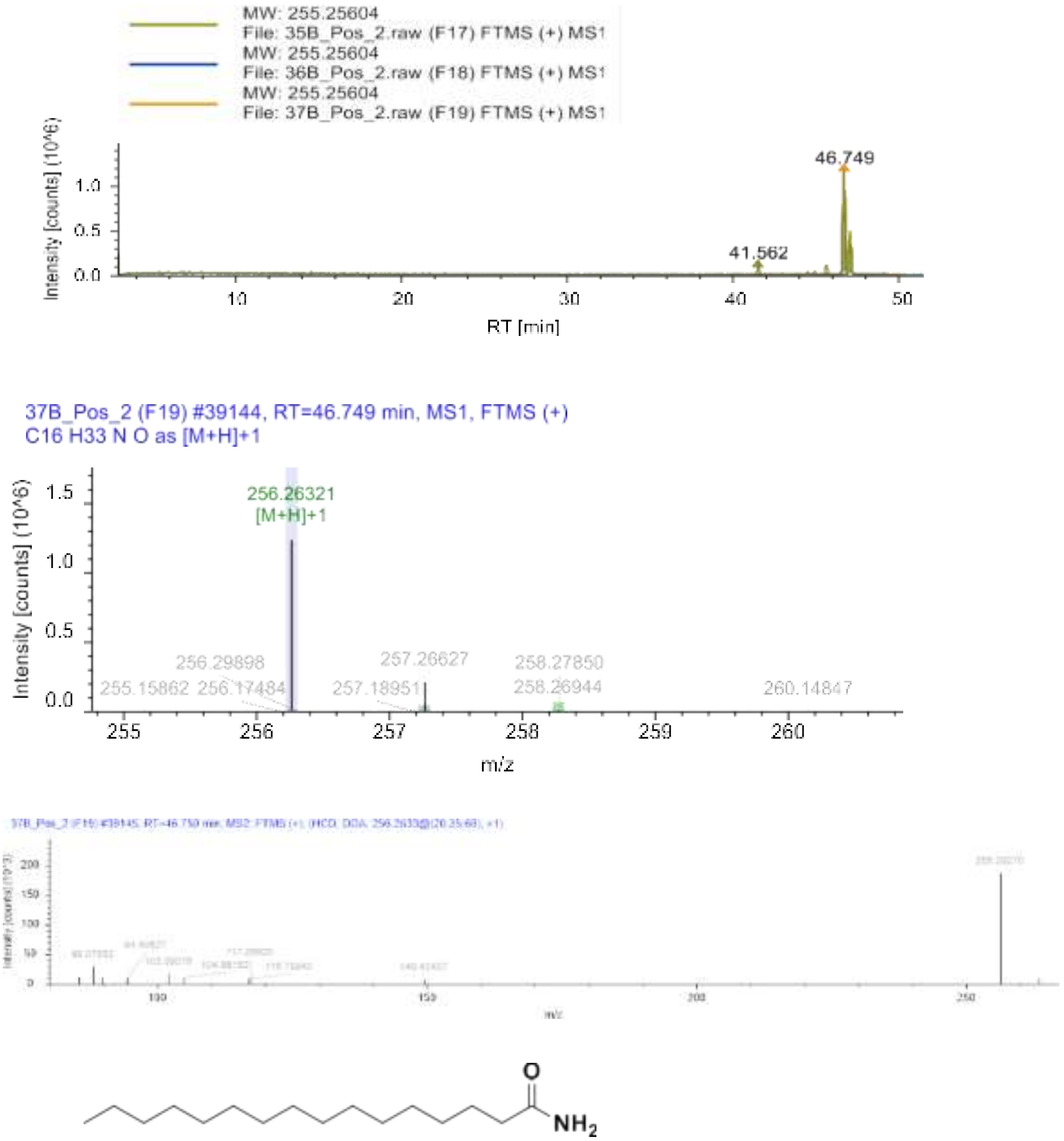

Figure S16: Retention time peak, fragmentation pattern and chemical structure of Hexadecanamide

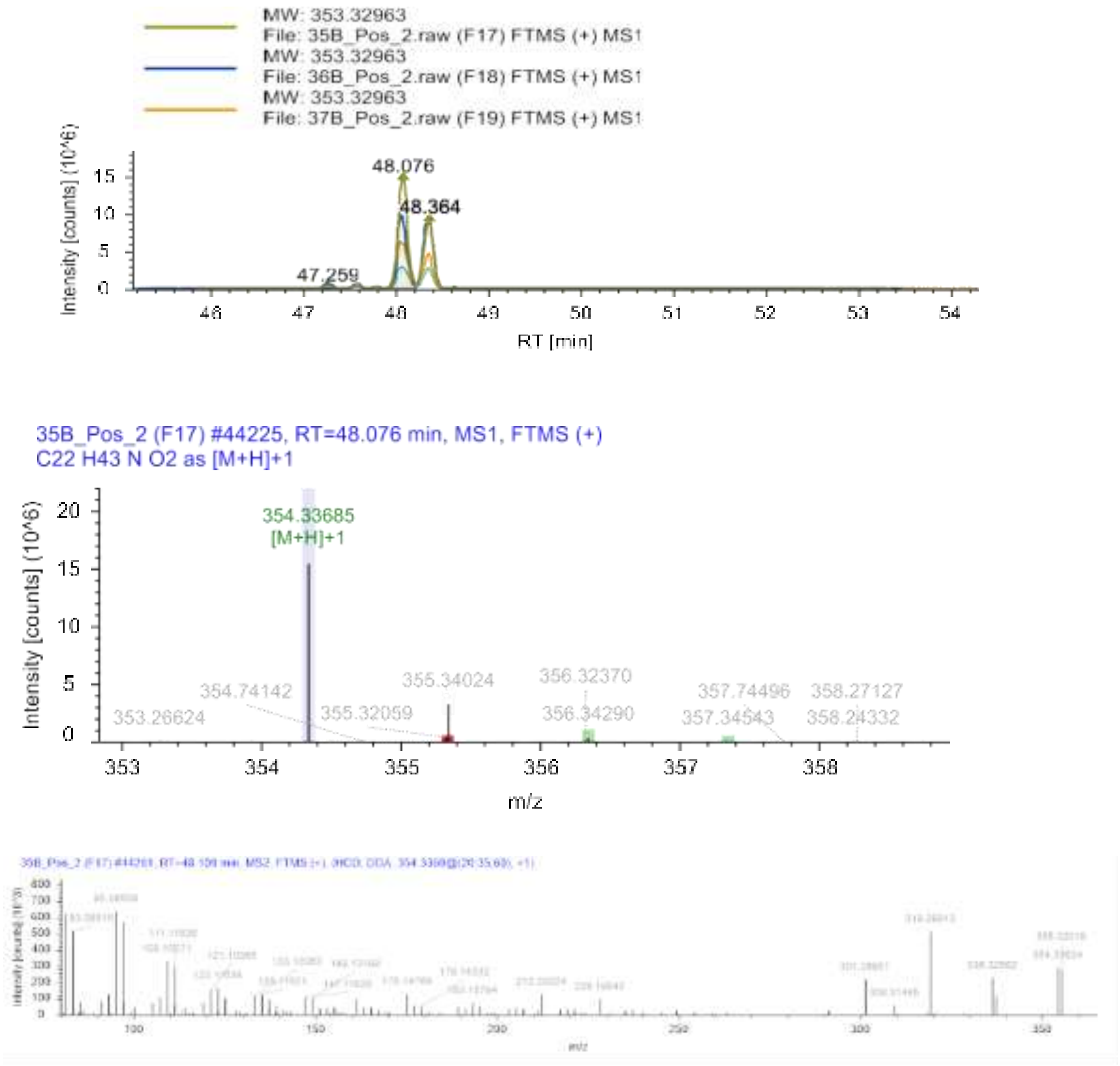

Figure S17: Retention time peak, fragmentation pattern and chemical structure of Unknown compound (not identified with library)

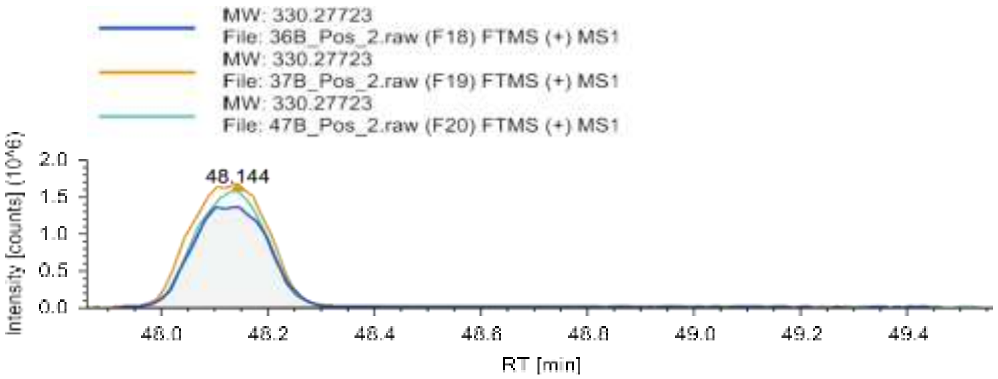

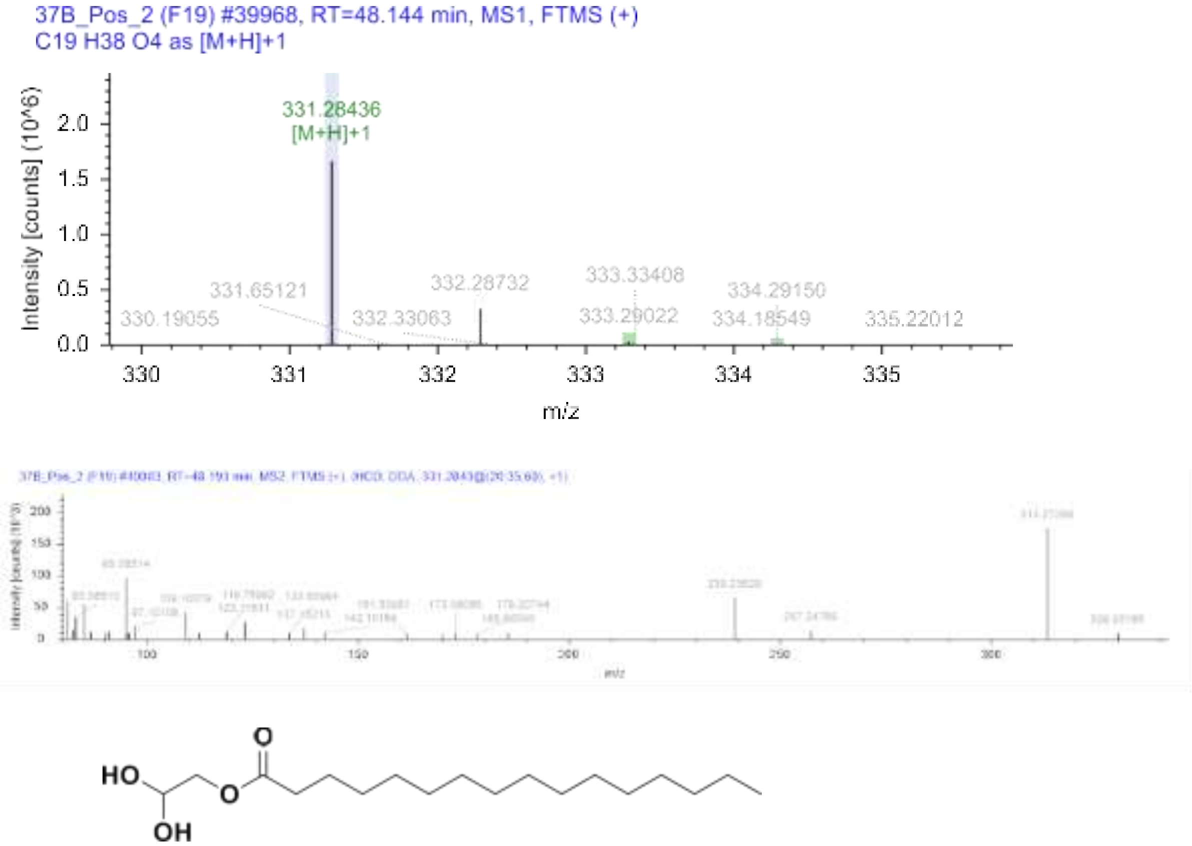

Figure S18: Retention time peak, fragmentation pattern and chemical structure of 1-Palmitoylglycerol

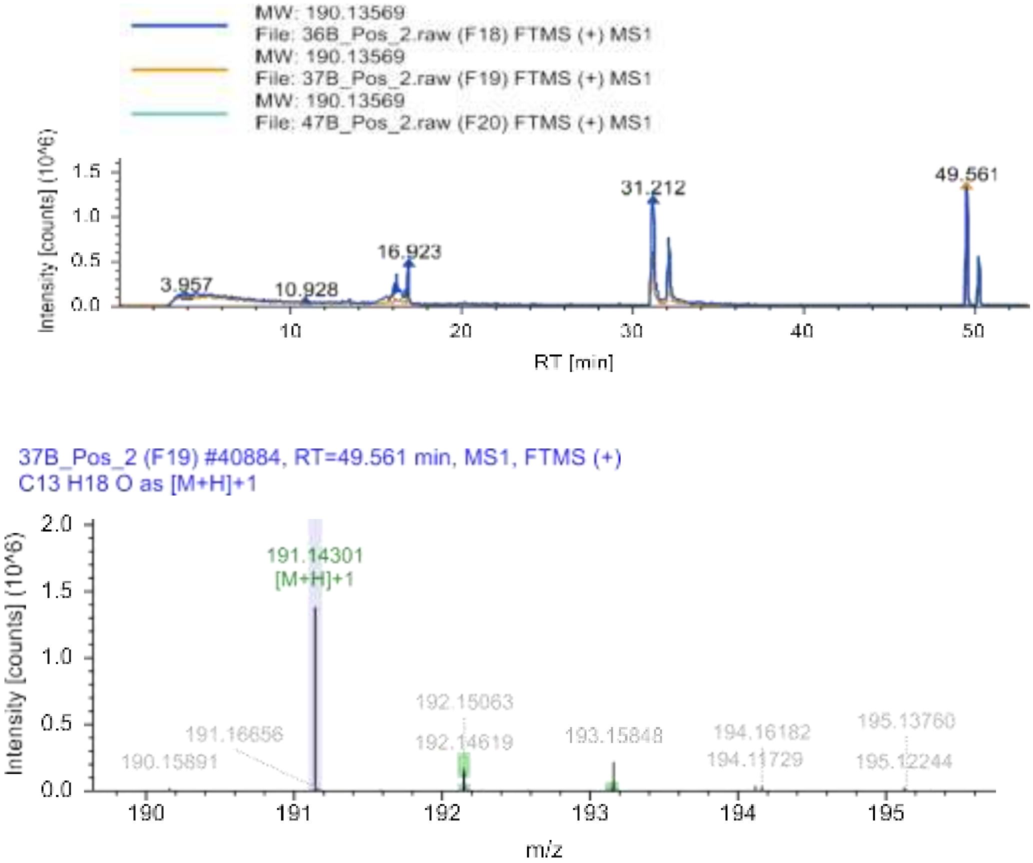

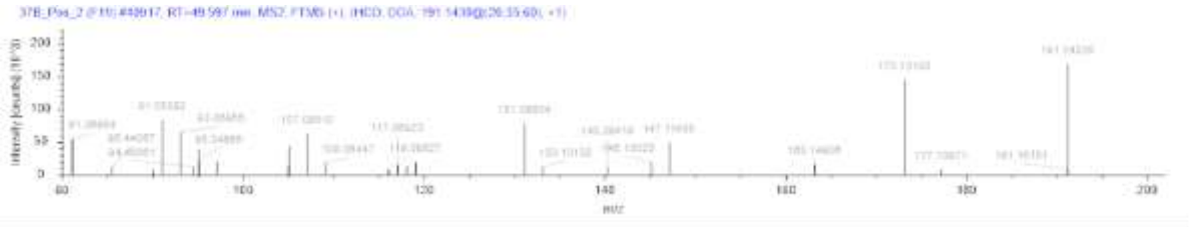

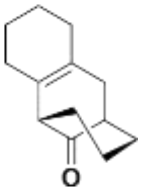

Figure S19: Retention time peak, fragmentation pattern and chemical structure of (1S)-Tricyclo[7.3.1.0~2,7~]tridec-2(7)- en-13-one

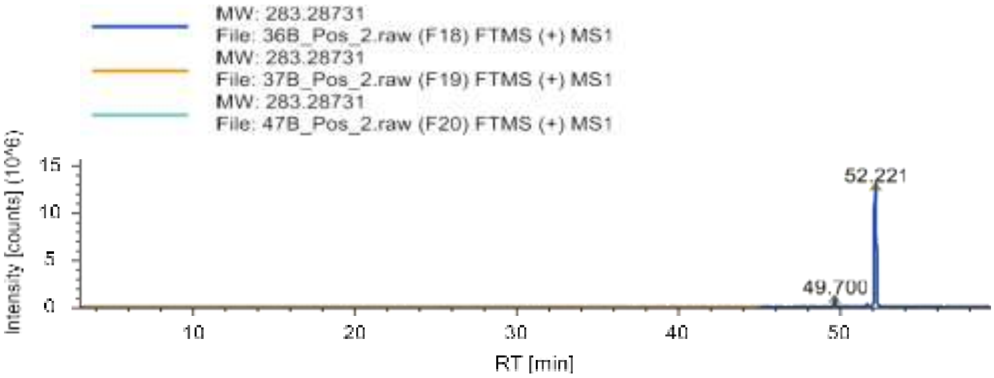

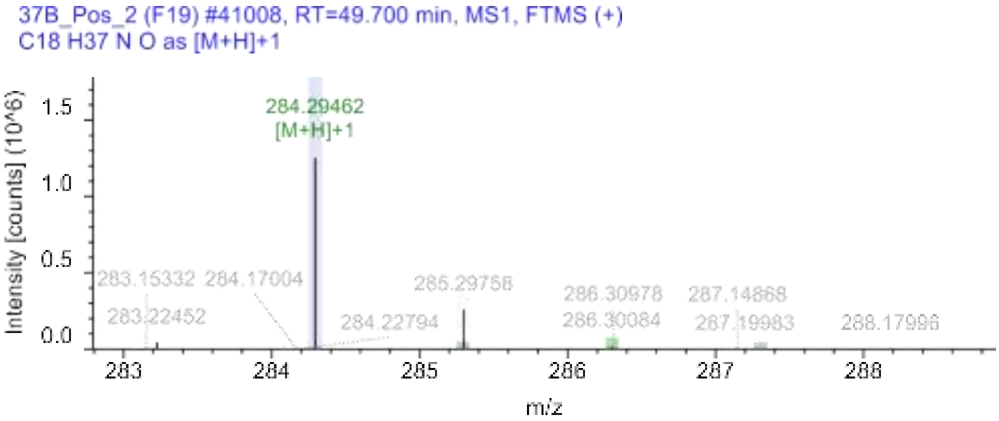

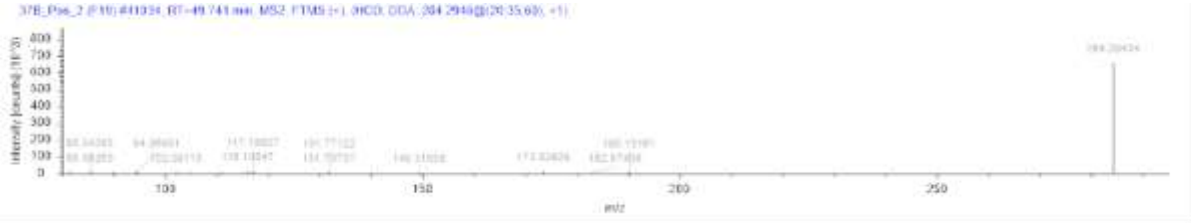

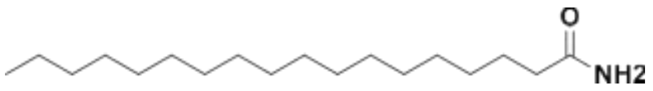

Figure S20: Retention time peak, fragmentation pattern and chemical structure of Stearamide

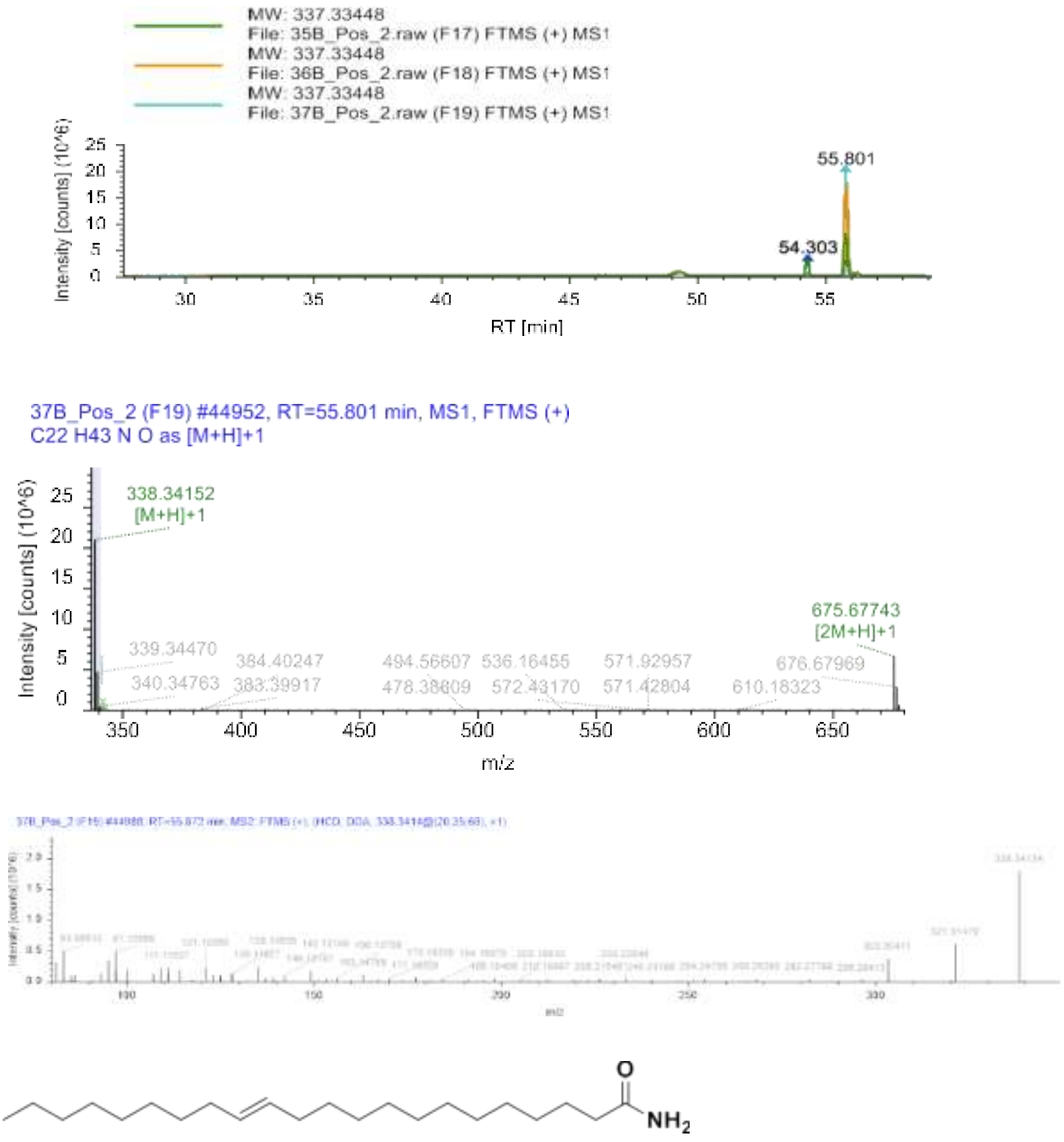

Figure S21: Retention time peak, fragmentation pattern and chemical structure of Erucamide

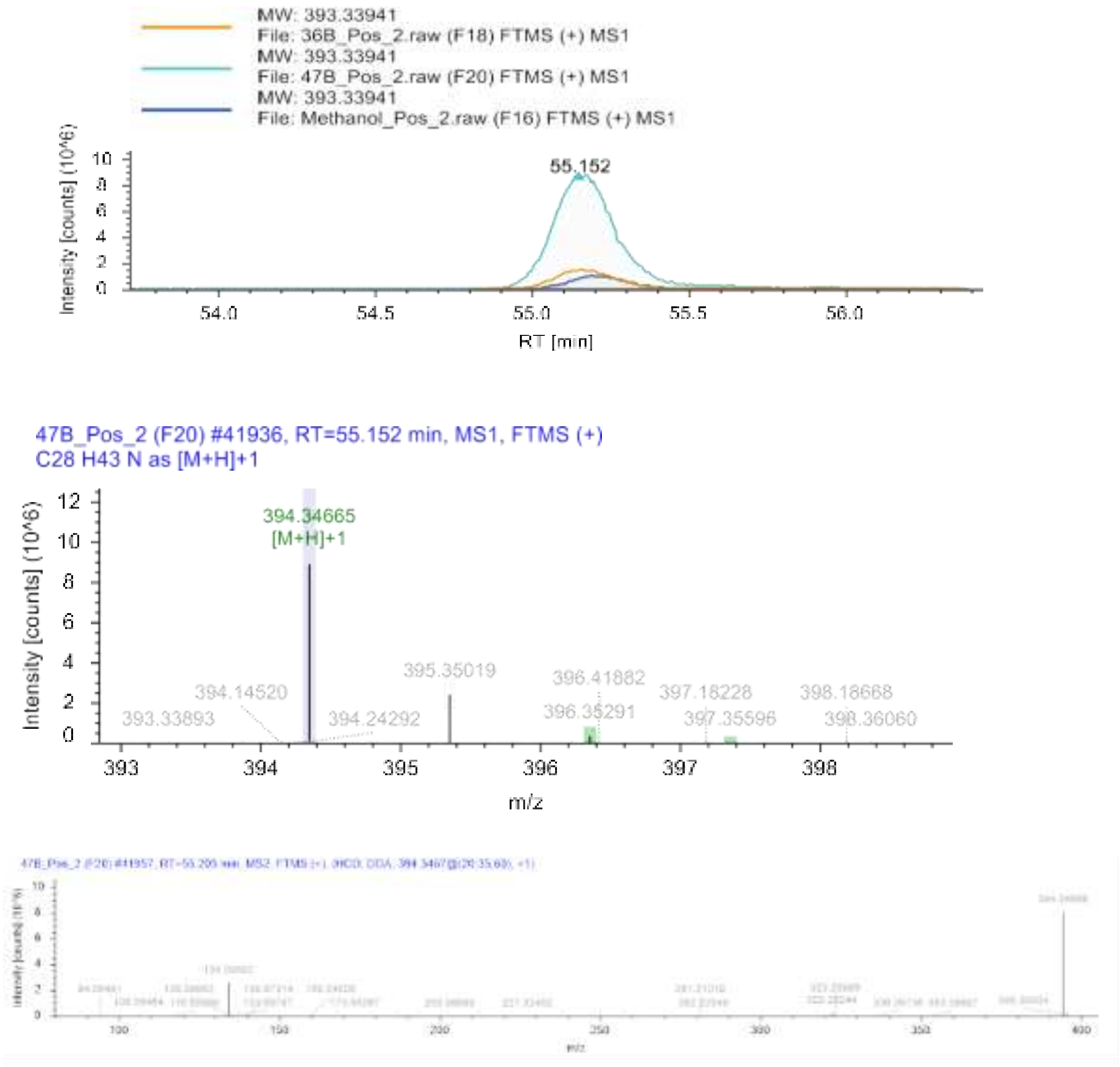

Figure S22: Retention time peak, fragmentation pattern and chemical structure of unknown compound (not identified with library)

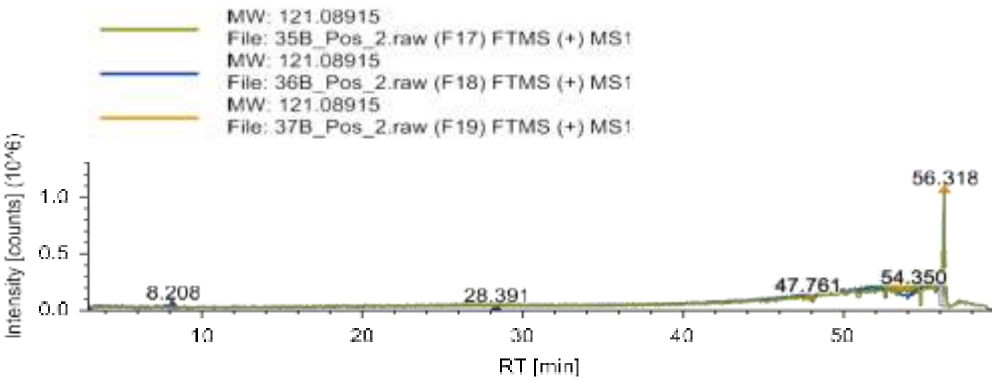

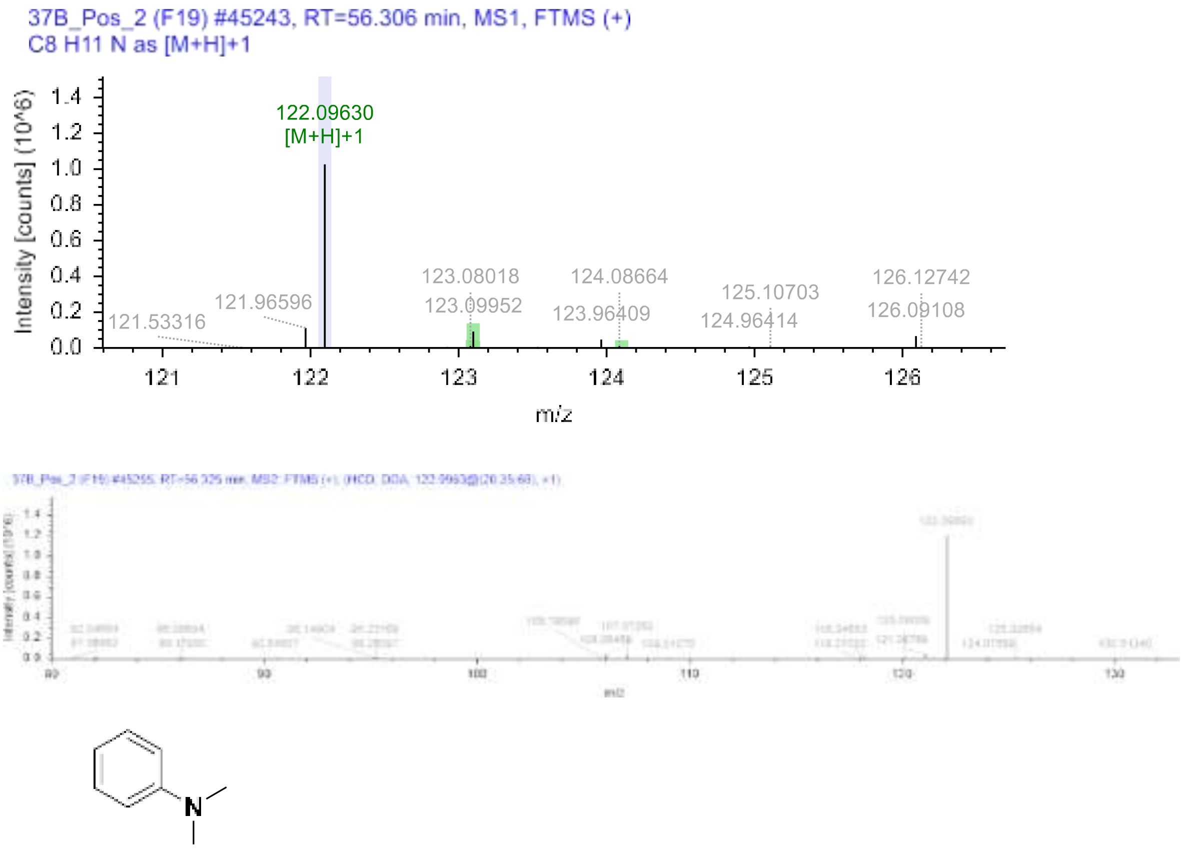

Figure S23: Retention time peak, fragmentation pattern and chemical structure of N,N-Dimethylaniline
